# Integrative pathway dissection of molecular mechanisms of moxLDL-induced vascular smooth muscle phenotype transformation

**DOI:** 10.1186/1471-2261-13-4

**Published:** 2013-01-16

**Authors:** George S Karagiannis, Jochen Weile, Gary D Bader, Joe Minta

**Affiliations:** 1 Department of Laboratory Medicine and Pathobiology, Faculty of Medicine, University of Toronto, Toronto, ON, M5S 1A8, Canada; 2 Department of Pathology and Laboratory Medicine, Mount Sinai Hospital, Toronto, ON, Canada; 3 Department of Molecular Genetics, University of Toronto, Toronto, ON, Canada; 4 The Donnelly Centre, University of Toronto, Toronto, ON, Canada

## Abstract

**Background:**

Atherosclerosis (AT) is a chronic inflammatory disease characterized by the accumulation of inflammatory cells, lipoproteins and fibrous tissue in the walls of arteries. AT is the primary cause of heart attacks and stroke and is the leading cause of death in Western countries. To date, the pathogenesis of AT is not well-defined. Studies have shown that the dedifferentiation of contractile and quiescent vascular smooth muscle cells (SMC) to the proliferative, migratory and synthetic phenotype in the intima is pivotal for the onset and progression of AT. To further delineate the mechanisms underlying the pathogenesis of AT, we analyzed the early molecular pathways and networks involved in the SMC phenotype transformation.

**Methods:**

Quiescent human coronary artery SMCs were treated with minimally-oxidized LDL (moxLDL), for 3 hours and 21 hours, respectively. Transcriptomic data was generated for both time-points using microarrays and was subjected to pathway analysis using Gene Set Enrichment Analysis, GeneMANIA and Ingenuity software tools. Gene expression heat maps and pathways enriched in differentially expressed genes were compared to identify functional biological themes to elucidate early and late molecular mechanisms of moxLDL-induced SMC dedifferentiation.

**Results:**

Differentially expressed genes were found to be enriched in cholesterol biosynthesis, inflammatory cytokines, chemokines, growth factors, cell cycle control and myogenic contraction themes. These pathways are consistent with inflammatory responses, cell proliferation, migration and ECM production, which are characteristic of SMC dedifferentiation. Furthermore, up-regulation of cholesterol synthesis and dysregulation of cholesterol metabolism was observed in moxLDL-induced SMC. These observations are consistent with the accumulation of cholesterol and oxidized cholesterol esters, which induce proinflammatory reactions during atherogenesis. Our data implicate for the first time IL12, IFN-α, HGF, CSF3, and VEGF signaling in SMC phenotype transformation. GPCR signaling, HBP1 (repressor of cyclin D1 and CDKN1B), and ID2 and ZEB1 transcriptional regulators were also found to have important roles in SMC dedifferentiation. Several microRNAs were observed to regulate the SMC phenotype transformation via an interaction with IFN-γ pathway. Also, several “nexus” genes in complex networks, including components of the multi-subunit enzyme complex involved in the terminal stages of cholesterol synthesis, microRNAs (miR-203, miR-511, miR-590-3p, miR-346*/miR- 1207-5p/miR-4763-3p), GPCR proteins (GPR1, GPR64, GPRC5A, GPR171, GPR176, GPR32, GPR25, GPR124) and signal transduction pathways, were found to be regulated.

**Conclusions:**

The systems biology analysis of the *in vitro* model of moxLDL-induced VSMC phenotype transformation was associated with the regulation of several genes not previously implicated in SMC phenotype transformation. The identification of these potential candidate genes enable hypothesis generation and in vivo functional experimentation (such as gain and loss-of-function studies) to establish causality with the process of SMC phenotype transformation and atherogenesis.

## Background

Atherosclerosis (AT) is a chronic inflammatory disease of medium and large arteries characterized by the accumulation of inflammatory cells, lipoproteins and fibrous tissue that lead to the formation of atherosclerotic plaques. It is the primary cause of heart attacks and stroke, and the leading cause of death and disability in developed countries [1-3]. AT is a multifactorial disease with genetic, environmental and lifestyle risk factors. A variety of atherogenic stimuli including hemodynamic shear stress, infections, lipids and proinflammatory cytokines induce endothelial cell dysfunction and permit the migration of mononuclear cells into the subendothelial space. This process is associated with the transformation of quiescent contractile smooth muscle cells (SMCs) to a proliferative and migratory phenotype. As a result of this transformation, SMCs migrate to the neointima where they produce an extracellular matrix that stabilizes the atherosclerotic plaque [4-9]. Lipids deposited in atherosclerotic plaques are derived largely from the lower-density lipoproteins [LDL, LDL-1, VLDL, beta-VLDL, and Lp(a)] of the blood [10,11]. 12/15- lipoxygenase and myeloperoxidase have been identified as lipid-oxidizing enzymes that are involved in the formation of biologically active oxidized lipids (cholesterol ester hydroperoxides). The accumulation of these oxidized lipids may initiate the proinflammatory activation of macrophages and SMCs in atherosclerotic lesions [12,13]. Mildly or minimally oxidized forms of LDL (moxLDL) activate both cell-mediated and humoral immune responses that perpetuate the chronic inflammatory reactions characteristic of atherosclerosis. The accumulation of cholesterol esters in macrophages and macrophage-like cells induce the release of pro-inflammatory cytokines, chemokines, reactive oxygen radicals, and matrix metalloproteinases [14-16].

Although the majority of foam cells, containing oxidized lipoproteins, in atherosclerotic lesions are derived from macrophages, SMCs also give rise to a significant number of lipid laden cells. SMCs exposed to atherogenic stimuli such as inflammatory cytokines, shear stress, moxLDL or reactive oxygen radicals or lipids [6,17-19] express high levels of a variety of lipid-binding membrane receptors including LDLR, VLDLR, LOX-1, CD36, type I and type II scavenger receptors, and CXCL16/SR-PSOX for cholesterol uptake [20]. Atherogenic cytokines such as IL-1α, TNF-α, and MCSF further upregulate the expression of LDLR and VLDLR [21]. The binding of moxLDL to these receptors then results in the accumulation of high levels of cholesterol and cholesteryl esters by the macrophages and SMCs, which then transform into foam cells in early fatty streak lesions. These changes characterize the initiation and progression of atherosclerosis and restenosis [19].

moxLDL has been shown to induce SMC transformation from the “contractile” phenotype to the “migratory, proliferative and synthetic” phenotype, central to intimal hyperplasia and atherogenesis [4,19]. Activated SMCs also produce cytokines such as PDGF, TGF-β and IFN, which contribute to the initiation and propagation of the inflammatory response of the vessel wall [18,19,22,23].

Recently, a number of investigators have used systematic approaches to investigate atherosclerosis. In these works, biopsies of stable and unstable plaques from symptomatic and asymptomatic patients as well as lesions in mouse models for AT have been examined. Gene expression profiles, pathways and molecular networks were analyzed, that underlie the formation of atherosclerotic plaques [24-31]. As a result, these studies have implicated many potential human atherogenic genes related to lipid homeostasis and have reported changes in the cytokine-induced immune and inflammatory responses as part of the pathogenesis of AT. Such studies have also underscored SMC dedifferentiation as a key process in the initiation and progression of AT.

Despite these advances, the molecular mechanisms of SMC transformation during initiation and progression of atherogenesis are not well-defined. However, the identification of early critical pathways involved in SMC transformation can provide insights into the mechanisms that underlie the pathogenesis of AT and cardiovascular diseases and could deliver potential targets for drug discovery. To facilitate such analyses, we have previously used oligonucleotide microarrays to analyze the genome-wide differential gene expression in quiescent primary human coronary artery SMCs induced with moxLDL for 3h and 21h [32]. This work uncovered several genes not previously implicated in the moxLDL-induced SMC phenotype transformation and described numerous functional categories of genes with altered gene expression.

Here, we significantly extended the original analysis of the resulting gene expression data using a number of pathway analysis tools - Gene Set Enrichment Analysis (GSEA) [33], Enrichment Map visualization [34], Ingenuity Pathway Analysis (IPA) and GeneMANIA. We found new, non-previously described functional themes and pathways, which may help elucidate the early and late mechanisms of moxLDL-induced SMC phenotype transformation and the onset and progression of atherogenesis. While the *in vitro* atherogenesis model involving moxLDL treatment of VSMC, particularly in the absence of endothelial cells and immune and inflammatory cells, is an oversimplified model of the complex process of atherogenesis, our systems analysis on the interactions of moxLDL and VSMC has uncovered several novel gene and pathway changes. These observations now permit hypotheses generation and *in vivo* functional testing (such as gain and loss-of-function studies) to establish causality with the process of SMC phenotypic transformation and atherogenesis.

## Methods

### Microarray analysis

The microarray analysis of moxLDL treated cells has been previously described [32]. Briefly, human coronary artery SMCs were purchased from Clonetics (Walkersville, MD) and cultured according to the manufacturer’s instructions and used between passages 4–7. Confluent SMC cultures were synchronized to quiescence by incubation for 48h in basal medium (SmBM) containing 0.5% FBS. The cells were then washed and incubated in SmBM*+*0.5% FBS in the absence or presence of unoxidized LDL or moxLDL (2 μg/ml) for 3h and 21h. The reactions were performed in quadruplicates. DNA-free mRNA was extracted from the cells and mRNA samples from corresponding cell cultures were pooled to reduce inter-sample variation. Biotinylated cRNA samples were hybridized to HG-U133A oligonucleotide Gene Chip arrays (Affymetrix, Santa Clara, CA). The data files from the arrays were analyzed using Affymetrix GeneChip® Operating Software (GCOS) version 1.0 (Affymetrix, Santa Clara, CA) to identify differentially expressed genes.

### Re-processing of gene expression data for Gene Set Enrichment Analysis

The originally published set of differentially expressed genes only contained those surpassing a threshold (at least 2-fold differentially expressed), however GSEA requires input of all genes ranked from most over-expressed to most under-expressed. To gather this information, we reprocessed the original Affymetrix HG-U133A CEL image data files using the Affy library of the Bioconductor package for the R programming language [35]. Three arrays exist in this experiment: control, treatment after 3h and treatment after 21h. Background correction and normalization was performed on the datasets using the RMA method [36]. This data was then reformatted for input into the GSEA software (GCT file format).

### Gene Set Enrichment Analysis (GSEA) based pathway analysis

Pathway enrichment analysis was carried out by searching for enriched gene-sets (e.g. pathways, molecular functional categories, complexes) in the early time point (3h) vs. control and the late time point (21h) vs. control using GSEA. It was not possible to use a statistical test to establish a gene ranking, as only gene expression data from one pooled set of samples was available for each experimental condition. Instead, a fold-change metric was used, computed by GSEA, comparing moxLDL-3h vs. Control and moxLDL-21h vs. Control. We used “gene set permutation” with 1000 permutations to compute p-values for enriched gene-sets, followed by GSEA’s standard multiple testing correction. We used GSEA’s built- in gene identifier (ID) conversion system to convert Affymetrix probeset IDs from the expression data matrices to gene symbols for analysis. We used an updated version (September 2, 2011) of a custom gene set collection previously used for pathway analysis [37] (http://baderlab.org/GeneSets). The collection comprises Gene Ontology annotations [38], as well as pathways from the HumanCyc [39], Kyoto Encyclopedia of Genes and Genomes (KEGG) [40], MSigDB [33], NCI Nature Pathway Interaction Database (PID) [41], NetPath [42] and Reactome [43] databases.

### Enrichment Map pathway analysis visualization

The resulting enrichment results were visualized with the Enrichment Map plugin for the Cytoscape network visualization and analysis software. We loaded GSEA results using a p-value cut-off of 0.005 and a q-value threshold of 0.1. In these maps, each gene set is symbolized by a node in the network. Node size corresponds to the number of genes comprising the gene-set. The enrichment scores for the gene-set are represented by the node’s color (red indicates up-regulation, blue represents down-regulation). The color of the node center indicates the enrichment score for the early time point (3h), and the node border color indicates the score for the late time point (21h). To intuitively identify redundancies between gene sets, the nodes are connected with edges if their contents overlap by more than 50%. The thickness of the edge corresponds to the size of the overlap. We used version 1.2 of the Enrichment Map software in Cytoscape 2.8.2.

### GeneMANIA

GeneMANIA (http://www.genemania.org/) finds other genes that are related to a set of input genes, using a very large set of functional interaction data [44]. Interaction data include protein and genetic interactions, pathways, co-expression, co-localization and protein domain similarity. We searched the GeneMANIA web site using differentially expressed genes underlying specific functional themes to find out how the genes interact with each other. The resulting sub-network containing our query genes and additional related genes helps interpret the mechanistic details of the functional themes we define.

### Ingenuity pathway analysis

We also used the commercial software Ingenuity Pathway analysis (Ingenuity® Systems; IPA) (http://www.ingenuity.com/) to identify enriched pathways and functional themes, as reported previously [45]. In particular, genes of interest, defined as those genes that were at least 2-fold differentially expressed, as reported in the original publication [32] were uploaded into the application as standard human gene symbols. Each gene identifier was mapped to its corresponding gene object in the Ingenuity Pathways Knowledge Base (IPKB). The IPKB, containing a large network of curated molecular interactions and pathways, was searched to find sub-networks enriched in genes of interest. A total of 77 and 205 genes were found to be network eligible for the 3h and 21h moxLDL experiments, respectively. Graphical representations of these sub-networks, containing direct and indirect molecular relationships, were generated.

## Results and discussion

### Overview of the integrative pathway analysis

**Figure 1:**
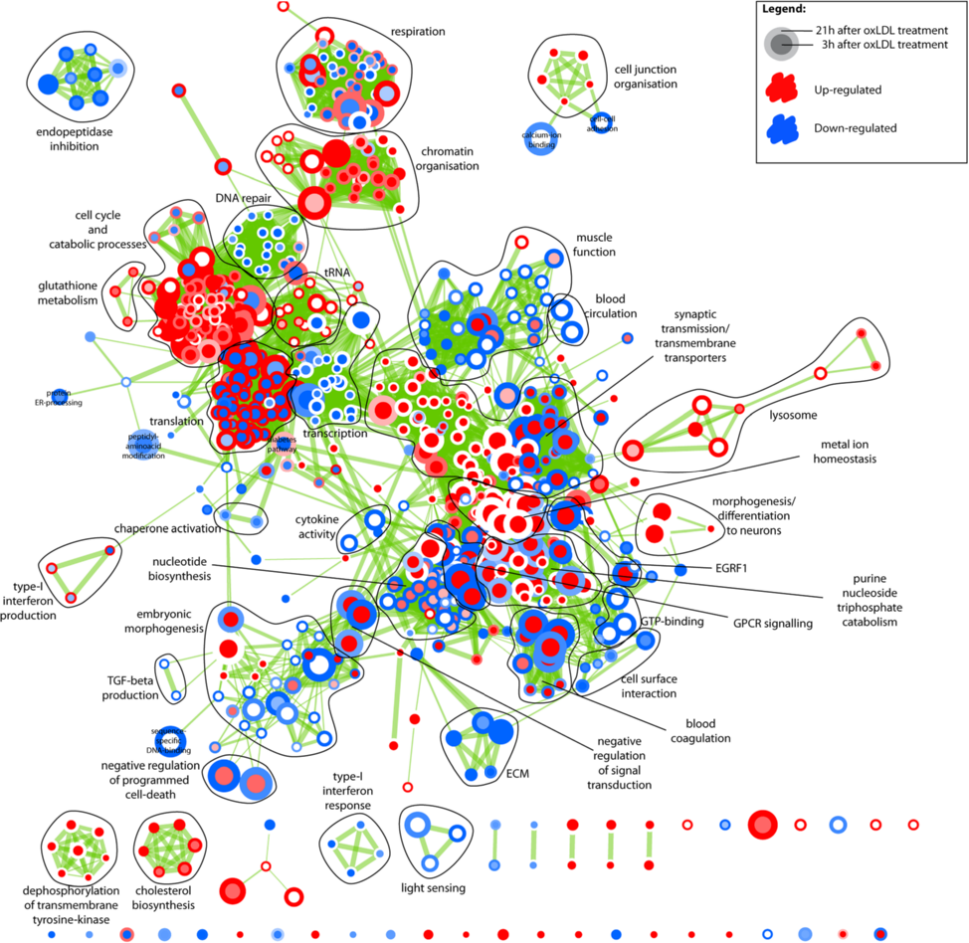
**Enrichment map for moxLDL, p=0.005, q=0.1; Nodes represent gene sets that are enriched at the top or bottom of the ranking of differentially expressed genes, as determined by GSEA, where the node size corresponds to number of genes in the set.** Edges indicate overlap between gene sets, where the thickness indicates the size of the overlap. Red indicates up-regulation, blue indicates down-regulation. The centre of the nodes corresponds to the early time point (3h), whereas the border of the node corresponds to the late time point (21h).

**Figure 2:**
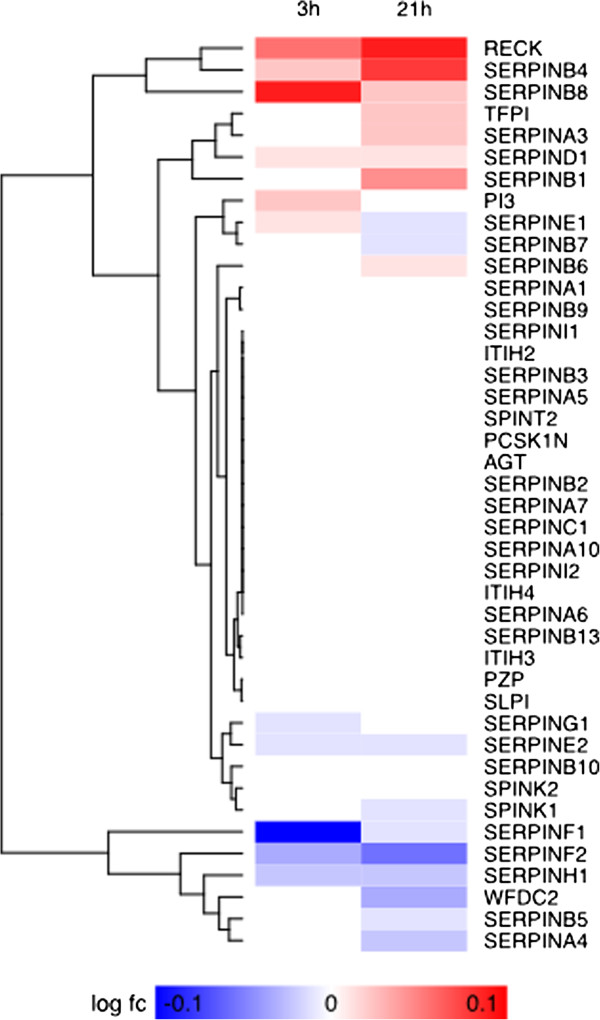
‘Endopeptidase Inhibition’ theme heatmap of representative SMC differential gene expression patterns induced by treatment with moxLDL at 3h and 21 h.

**Figure 3:**
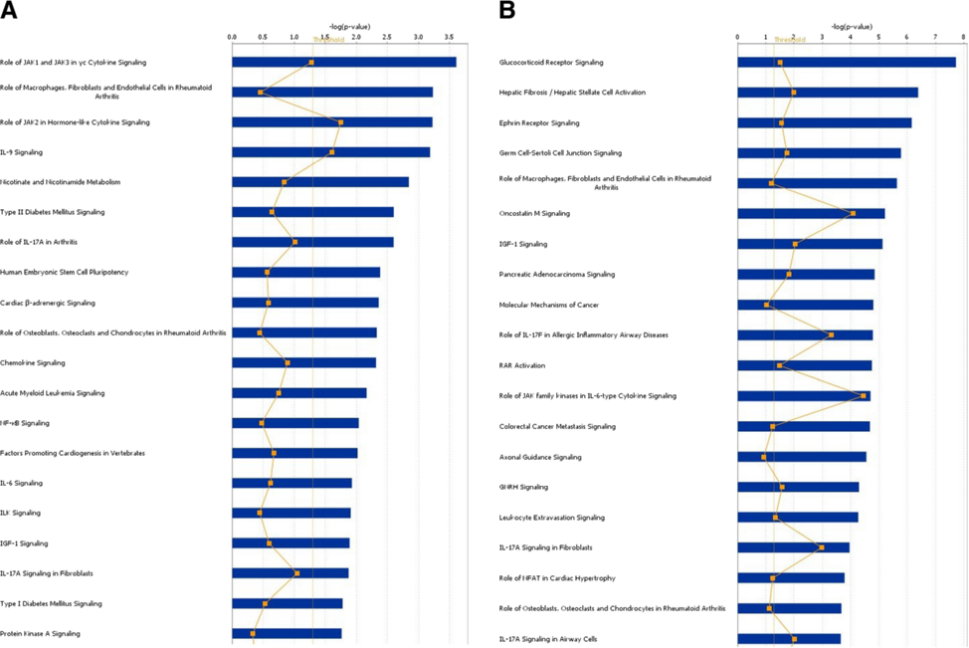
**Canonical Pathways from IPA.** Bars correspond to the top 20 Canonical Pathways that surpassed the Ingenuity statistical threshold (orange squares) using the Fisher’s exact test in the 3h (3**A**) and 21h (3**B**) moxLDL experiments, respectively.

Our GSEA-based analysis was summarized with the visualization of an enrichment map depicting a variety of molecular processes, here termed “functional themes” (Figure 1). These themes are listed in Additional file 1: Table S1, with a brief summary of their expression behavior at both 3h and 21h time points, following moxLDL treatment. We observed an up-regulation of genes in functional themes related to proliferation, cell migration, ECM production, cholesterol biosynthesis and protein translation (Figure 1). These observations are consistent with the hallmarks of SMC transformation. The differential gene expression patterns for specific functional themes of interest, mostly non-reported in the SMC transformation, are also shown as heat maps. For instance, it is evident that the “endopeptidase inhibition” theme is significantly down-regulated (Figure 1, Additional file 1: Table S1) and several members of the Serpin family of protease inhibitors are significantly down-regulated at 21h, as shown in the associated heat map (Figure 2). This down-regulation suggests increased proteolytic activity during SMC transformation. To our knowledge, endopeptidase activity has not been reported to play any role in SMC transformation. Other heatmaps of interest are shown in Additional file 2: Figure S1. In a complementary fashion, the investigation of canonical pathways at 3h (Additional file 3: Table S2) and 21h (Additional file 4: Table S3) using IPA, revealed enrichment in specific signaling and metabolic pathways. The 20 most significant ones were selected for further study (Figures 3A and 3B, respectively). Of note, JAK/STAT, Interleukin and IGF-1 signaling pathways, were deregulated at both time points. The dataset was further organized to predict how moxLDL treatment on SMCs might influence the cross-talk among interacting proteins. We identified eight major networks involving differentially expressed genes (4 networks at 3h; 4 networks at 21h). Interestingly, certain molecules in these networks (see below for details) were found to nucleate clusters of protein-protein interactions that may act as organizational “hubs” and additionally verified certain functional themes of the GSEA-based pathway analysis.

### In-depth pathway analysis of specific molecular themes of interest

#### Cholesterol biosynthesis

**Figure 4:**
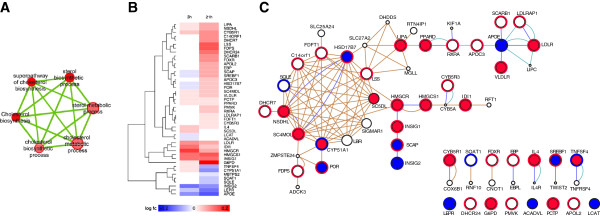
**Cholesterol biosynthesis theme analysis.** (**A**) Cholesterol biosynthesis theme from enrichment map. (**B**) Cholesterol biosynthesis-associated heatmap. (**C**) Network of interactions among moxLDL-SMC cholesterol metabolism related genes, as retrieved by the GeneMANIA website, colored by gene expression at 3h (node center) and 21h (node border). Red indicates up-regulation, blue indicates down-regulation and white indicates no differential expression or no expression data available. Circles represent genes and connecting lines represent interactions between genes. The network was generated using a GeneMANIA query of 43 cholesterol synthesis related genes (large circles) differentially expressed at either 3h or 21h. GeneMANIA retrieved known and predicted interactions between these genes and added extra genes (smaller white circles) that are strongly connected to query genes (we used the default setting of 20 additional connecting genes). Light blue lines indicate pathway interactions from the Reactome pathway database, dark blue lines indicate experimentally determine physical interactions, from various protein interaction databases included in GeneMANIA, and brown lines indicate predicted interactions, mostly from the I2D database of protein interactions predicted from experimentally determined physical interactions in other species. GeneMANIA advanced settings were used to search only physical, pathway and the default set of predicted networks. We excluded co-expression, co-localization and genetic interaction networks from the search to focus the analysis on higher confidence physical and pathway interactions.

Since the molecular mechanisms for SMC phenotype transformation during AT have not yet been clearly delineated, we initially examined the cholesterol biosynthesis theme (Figure 1) in SMC stimulated with moxLDL for 3h and 21h in detail (Figure 4A). Eight cholesterol synthesis-related genes were up-regulated in 3h with LDLR, IDI1, HMGCS1, INSIG1 moderately up-regulated and HMGCR highly up-regulated. Seven genes were down-regulated with INSIG2 and APOE being the most strongly decreased ones (Figure 4B). A GeneMANIA network analysis for interactions among the gene products suggested an initiation of cholesterologenesis with HMG-CoA synthetase (HMGCS), conversion of acetyl-CoA and acetoacetyl-CoA to 3-hydroxy-3-methylglutaryl- CoA (HMG-CoA) and subsequent HMG-CoA reductase (HMGCR) catalysis of the rate-limiting step in cholesterol biosynthesis by converting HMG-CoA to mevalonate (Figure 4C). INSIG1 accelerates the degradation of HMGCR in the proteasome [46]. SCAP binds and retains INSIG1 in the ER and the binding of INSIG1 to SREBP1 and 2 facilitates SCAP-mediated transport of SCAP-SREBP complexes to the Golgi complex for degradation [47]. In the presence of sterols, INSIG2 regulates lipid synthesis by blocking the proteolytic activation of SREBPs by SCAP [48]. The down-regulation of SCAP, SREBF2 and INSIG2 and up-regulation of INSIG1, HMGCS1 and HMGCR in moxLDL-SMC indicates the initiation of cholesterol synthesis in the 3h moxLDL-SMC cells. 

Cholesterol metabolism genes in 21h moxLDL-SMC were more robustly regulated with 26 genes up-regulated and 7 genes down-regulated. The most highly upregulated genes were G6PD, INSIG1, HMGCS1, FDPS and LSS and the most strongly down-regulated genes were APOE, LEPR, INSIG2, CYP51A1 and TNSF4 (Figure 4B). GeneMANIA network analysis indicated that genes encoding enzymes essential for the sequential enzymatic conversion of Acetyl-CoA and Acetoacetyl-CoA to cholesterol were all up-regulated in moxLDL-SMC (Figure 4C). The analysis also showed multiple interactions among the enzymes (including FDFT1, NSDHL, HSD17B7, LSS, SOLE, SC5DL, SC4MOL and CYP51A1) involved in the sequential conversion of farnesyl pyrophosphate to squalene, oxidosqualene, lanosterol and finally cholesterol and suggested that these enzymes are hub proteins or function as a multi-subunit complex.

The ER-bound INSIG-SCAP-SREBP complex is the most important sensor of sterol levels. At high cholesterol levels, the complex is retained in the ER, but at lower levels the SCAP-SREBP enters transport vesicles [47]. In the Golgi, SREBP undergoes two steps of proteolysis, releasing a soluble transcription factor that regulates many genes associated with cholesterol and lipid metabolism. This leads to increased synthesis of cholesterol and LDL receptors. A switch-like response that helps to maintain cellular cholesterol in a narrow range has been demonstrated in the ER. It is currently unclear whether the sharp transition is due to cooperative protein-protein interactions between SCAP molecules or an abrupt change in the chemical activity of cholesterol in the ER membrane when it crosses a threshold value [49]. It has been proposed that the level of expression of INSIG1 protein can influence the cholesterol-dependent transition point, and reduction of cholesterol levels leads to proteasomal degradation of INSIG1, which sensitizes cells to cholesterol depletion [46]. In our study, INSIG-1 is highly expressed at 21h and thus we predict sustained cholesterol synthesis would occur.

PDGF has been shown to regulate ABCA1 expression in SMC [50]. However in our study, both ABCA1 and ABCG1 were not expressed in moxLDL-treated SMC at 3h and 21h, in spite of an increased PDGF expression and cholesterol biosynthesis. We propose that the lack of ABCA1 and ABCG1 in moxLDL-treated SMC, would result in impaired cholesterol efflux leading to its accumulation in SMCs during atherogenesis. This finding is thus analogous to the observed down-regulation of ABCA1 and ABCG1 transporters in lipid laden macrophages which results in a dysregulated reverse cholesterol transport pathway that enhances lipid accumulation and “foam cell” formation in moxLDL-treated macrophages [51-55].

The ER contains acetoacetyl CoA thiolase (ACAT1), the enzyme responsible for esterifying excess cholesterol for storage in lipid droplets [56]. Cholesterol ester storage and accumulation as oil droplets in microsomes occurs during cholesterologenesis and may contribute to formation of fatty streaks. NAD(P)H dehydrogenase-like protein (NSDHL), a C4 demethylase that is involved in the removal of C-4 methyl groups from the cholesterol precursor lanosterol, is localized to the surface of the ER. It also accumulates on the surface of lipid droplets that function as intracellular storage compartments for neutral lipids and cholesterol esters and participates in the regulation of cellular cholesterol content [57]. The up-regulation of NSDHL in moxLDL-SMC may therefore play a role in the accumulation of cholesterol in moxLDL-SMC.

Cholesterol metabolism was tightly regulated in 21h moxLDL-SMC, judging by the differential regulation of the network of LDLR, LDLRAP1, LIPA, RXRA, APOC3 and APOL2 genes (Figure 4C). LDLRAP1 is required for internalization of the LDL-LDLR complex in endocytic vesicles [58]. Lysosomal acid lipase **(**LIPA) has been reported to play an important role in cellular metabolism by releasing cholesterol, which in turn suppresses further cholesterol synthesis and stimulates esterification of cholesterol within the cell. ApoE knock-out mice spontaneously develop atherosclerosis. However, this effect is counteracted by the retinoid X receptor (RXRA) in the same model [59]. APOC3 inhibits the catabolism and hepatic uptake of apoB-containing lipoproteins and enhances the catabolism of HDL particles, as well as the adhesion of monocytes to vascular endothelial cells and activates inflammatory signaling pathways [60]. The up-regulation of APOC3 in moxLDL-SMC would inhibit cholesterol clearance via HDL. Interestingly, the observations of up-regulation of LDLR, LDLRAP1, INSIG1, SCAP, LIPA, RXRA, NSDHL, APOC3 and APOL2 and the down-regulation of INSIG2 and APOE in moxLDL-SMC further suggest a dysregulation of cholesterol metabolism and clearance in moxLDL-SMC, a situation that favors foam cell formation. APOL2 has not been reported to be expressed in neointima or the media but is up-regulated in HUVECs following prolonged stimulation with TNF-α [61-63].

To date, statins are used therapeutically to inhibit *de novo* hepatic cholesterol synthesis to lower the levels of plasma LDL-cholesterol, the major risk factor for atherosclerosis and coronary heart disease. Inhibition of HMGCR conversion of HMG CoA to mevalonic acid results in an inhibition of the synthesis of several non-sterols such as dolichols and ubiquinone and contributes to the side effects observed in patients on statin therapy. Consequently, attention has been directed towards enzymes such as squalene synthetase, squalene epoxidase and oxidosqualene cyclase, which are involved in cholesterol synthesis beyond farnesyl pyrophosphate as potential targets. Preclinical studies with oral bioavailable inhibitors have demonstrated the potential of squalene epoxidase inhibitors as hypocholesterolemic agents, however high circulating levels of squalene epoxidase inhibitors are believed to be responsible for dermatitis and neuropathy observed in the participants [64]. To the best of our knowledge, the hypocholesterolemic effects of inhibitors of the enzymes involved in post-squalene cholesterol biosynthesis have not yet been reported. These enzymes [including LSS, SC5DL, CYP51A1, SC4MOL, NSDHL, HSD17B7 and C14orf1 (Figure 4C)] exhibit extensive physical interactions with each other, suggesting they may form a scaffold and act via a multistep multi-enzyme process. Inhibition of any one of the enzymes may destabilize the complex and inhibit cholesterol formation. They, thus, could be considered potential targets for the design of hypocholesterolemic drugs for therapeutic intervention of SMC phenotype transformation and atherogenesis.

The significance of moxLDL-mediated induction of cholesterol synthesis in SMC phenotype transformation is believed to be related to the proinflammatory properties of atherogenic LDL particles. According to the “oxidation hypothesis of atherogenesis”, specific proinflammatory oxidized phospholipids that result from the oxidation of LDL phospholipids containing arachidonic acid are largely generated by potent oxidants produced by the lipoxygenase and myeloperoxidase pathways. These so-called mildly or minimally oxidized LDL and their active components, such as polyoxygenated cholesteryl ester hydroperoxides, are recognized by the innate immune system via Toll-like receptor (TLR) activation (such as TLR4). This leads to the recruitment of spleen tyrosine kinase (Syk), cytoskeletal rearrangements and macropinocytosis which in hyperlipidemic environments leads to excessive lipid accumulation in monocytes, macrophages and SMC in vascular lesions, foam cell formation, vascular inflammation and ultimately the development of atherosclerotic plaques [65,66]. TLR engagement stimulates multiple signaling pathways, including PI3-K/Akt and p38/p42/p44 MAPKs, which activate transcription factors (e.g. NFkB, IRFs, STAT1, STAT3, AP-1). The activation of these pathways also activates the expression of proinflammatory cytokines (e.g. IL1α TGFβ, MCP-1, IFNγ) and growth factors (e.g. PDGF, IGF, EGF, FGF) involved in mitogenesis of VSMCs [67-76]. The various functional themes and pathways that we have analyzed substantiate these observations and suggest further details about the molecular mechanisms of SMC phenotype transformation.

#### Inflammatory cytokines and growth factors

**Figure 5:**
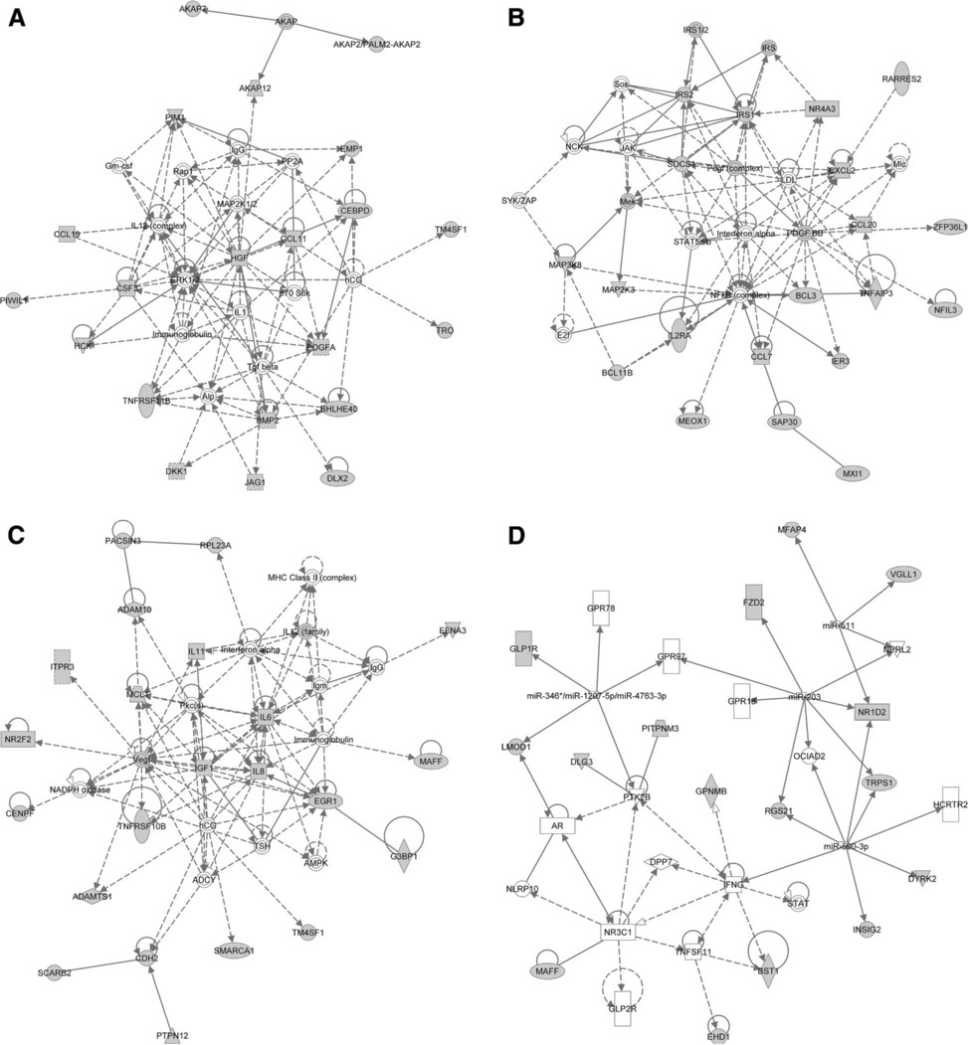
**Cytokine and growth factor theme analysis.** IPA networks from 3h (**A**, **B**, **D**) and 21h (**C**) experiments, involving cytokines and growth factors as molecular hubs. Genes/proteins are illustrated as nodes and molecular relationships as connecting lines between two nodes (direct relationships as normal lines; indirect relationships as dashed lines). Molecular relationships are supported by at least one literature reference, or by canonical information stored in the IPKB. Grey nodes represent genes of interest, while white nodes represent hubs that were added by the IPA algorithm to connect a set of genes of interest.

GSEA-based and IPA analysis found large clusters of cytokines, including IL-1, IL-12, CSF-3, TGF-β, PDGF and HGF, grouping many of the differentially expressed genes of the dataset (Figure 1, Figure 5A). Other networks identified “hubs” belonging mainly to the IFN-α, PDGF, NF-kB, VEGF and JAK/STAT signaling pathways (Figure 5B). Such networks were also found in the 21h treatment experiments, where members of the interleukin family of proteins nucleated clusters of signaling molecules regulating cell growth, proliferation and migration (Figure 5C). Cytokines and growth factors, including Interleukins (IL-6, IL-1β), TGF-β, PDGF, HGF, IGF-1 and members of the IFN family, have been shown to activate signal transduction cascades that trigger remodeling of the cytoskeleton and change cell-to-matrix adhesion [77]. Hepatocyte growth factor (HGF), previously linked to the regulation of cell motility and migration especially in cancer and atherosclerosis [78], nucleated a network with members of the MAPK family. In another network, IFN-γ was the major molecular “hub” (Figure 5D). IFN-γ, known to be released at sites of inflammation and in large amounts in the plaque, induces vasodilation and synthesis of NO by SMCs, which in turn contributes to hyperemia of inflammation. IFN-γ-induced NO synthesis by SMCs may also be involved in the regulation of vascular tone and proliferation of SMCs [79,80]. To the best of our knowledge, the activation of IL12, IFN-α, HGF and VEGF signaling pathways in SMC undergoing phenotype transformation has not been reported. In a complementary fashion, canonical pathways belonging to these networks were also enriched in our dataset, as seen in Figures 3A and 3B. 

MicroRNAs (miRNAs) have recently been implicated in the regulation of atherosclerosis and lipoprotein metabolism, by affecting endothelial integrity, macrophage inflammatory response to atherogenic lipids, vascular smooth muscle cell proliferation, and cholesterol synthesis [81]. We found that certain miRNAs (miR-203, miR-511, miR-590-3p, miR-346*/miR-1207-5p/miR-4763-3p) serve as organizational hubs of several signal transduction pathways in one of our IPA networks (Figure 5D). Since miRNAs are implicated in inflammatory processes that accompany heart failure, AT, coronary artery disease, obesity and diabetes [82], we further investigated these pathways. Many of the identified miRNAs, along with clusters of deregulated proteins were, indeed, highly connected to the IFN-γ pathway in the same molecular network (Figure 5D). Interestingly the JAK/STAT, MAPK and IGF signaling pathways, which have been shown to play clearly defined roles in AT pathogenesis [83-85], served as major intracellular mediators of the cytokine pathways in the generated molecular networks. Recent integrative approaches demonstrating a plethora of IFN-γ-regulated mRNAs and targeted mRNAs [86], coupled with our observation of miRNAs in the IFN-γ-dominated molecular network suggest that inflammatory signaling may be regulated through non-classical miRNA-related cytokine pathways, beyond the classical JAK/STAT and MAPK pathways.

#### G-protein coupled receptors (GPCRs)

VSMC migration involves a dominant plasma membrane leading lamellae, or leading edge, protruding from the cell to make contact with an extracellular substrate. Binding is accomplished via integrin transmembrane receptors that enable the formation of focal complexes and secure focal adhesions. An intracellular signal transduction cascade, involving G-protein and tyrosine-kinases, results in the alignment of actin filaments and a myosin contraction within the leading edge. Focal adhesions are subsequently disengaged over the remainder of the cell surface, and contractile forces propel the cell forward in the direction of the anchoring leading edge [87-91]. Thus, VSMC migration is predominantly regulated by two receptor-coupled systems, GTP-binding protein (G-protein)-coupled and tyrosine kinase-coupled proteins. Signal transduction pathways from these two systems appear to intersect as signals are transmitted.

**Figure 6:**
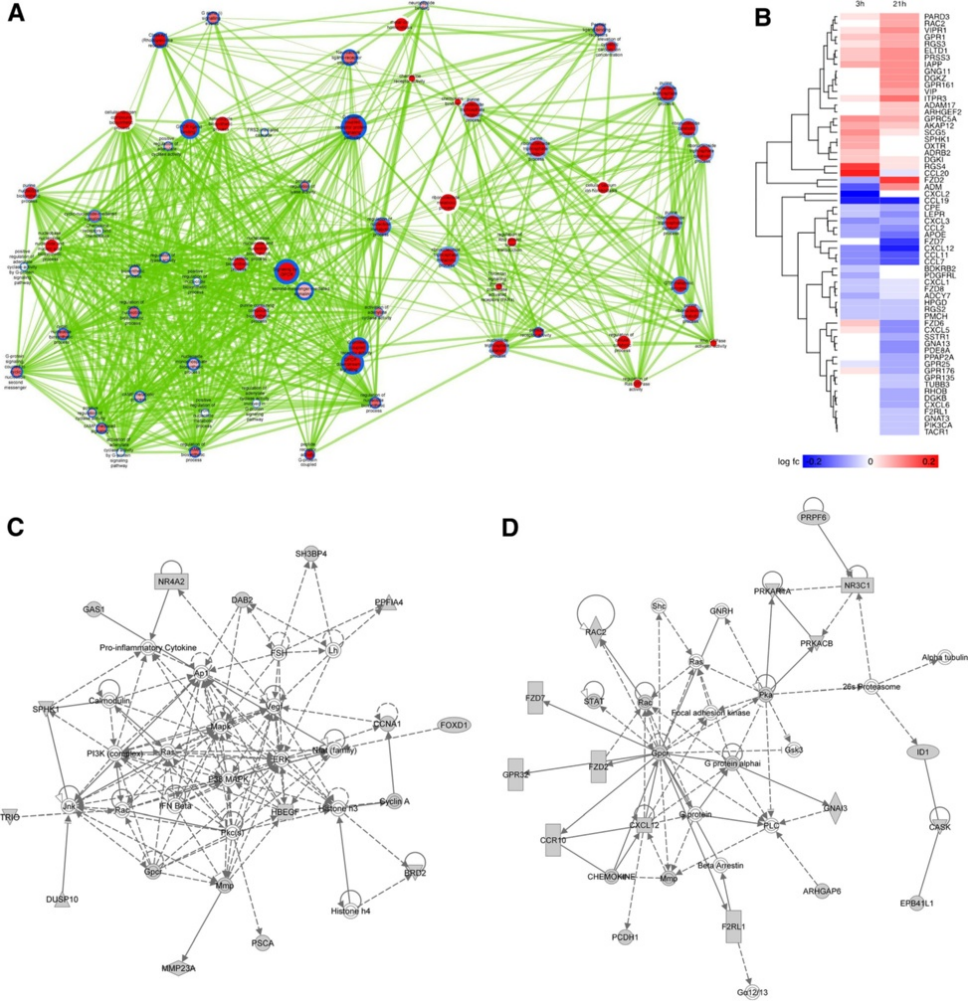
**GPCR signaling theme analysis. **(**A**) GPCR theme from enrichment map. (**B**) GPCR - associated heatmap. (**C**)GPCR-associated IPA network (3h) (**D**) GPCR-associated IPA network (21h). For color coding interpretation, please refer to Figures 1 &5.

To-date the mechanism of action of GPCRs in SMC migration has not been well delineated. The differential expression of the member genes of the GPCR theme (Figure 1, Figure 6A) is shown in the associated heat map (Figure 6B). Many of these genes (e.g. GPR1, GPR64, GPRC5A, and GPR171), expected to be involved in regulating SMC transformation, are up-regulated, whereas Frizzled6, Frizzled8, GPR176, GPR32, GPR25, and GPR124 are down- regulated. Frizzled2 is down-regulated at 3h but strongly up-regulated at 21h. The receptors encoded by these genes are specific to different signaling molecules. The fact that one group of receptors seems to be produced increasingly at the expense of a second group could indicate a shift of the cell’s responsiveness to different sets of signals. The most strongly up- and down-regulated genes in the GPCR functional theme encode various chemokines (CCL19, CCL20, CXCL2, and CXCL12). These observations suggest that GPCRs could regulate cell migration and trafficking of immune cells as well as VSMC early in the course of mox-LDL treatment (~3h) and that their effects might not be retained at later events (~21h) (Figure 1, Figure 6B, Table 1). Additionally, an IPA network of particular interest for the 3h treatment experiment is shown in Figure 6C, where GPCRs interact indirectly with members of the MAPK signaling pathway, and thus may be regulating important biological processes, such as cell growth and proliferation, migration and differentiation. Moreover, the GPCR family also acts as an organizational hub in the 21h experiment (Figure 6D), during which the enrichment map shows a significant down-regulation of the GPCR functional theme (Figure 6A), as already described. IPA proposed that GPCRs regulate important signaling pathways, several of which are revealed in our molecular networks (Figure 6D): For example, (a) Rac, is a member of the Rho family of proteins. Rho kinases have been widely demonstrated to be up-regulated in activated SMCs by inflammatory stimuli [90], (b) CXC-motif type chemokines, which regulate chemotactic responses, may participate in the recruitment of inflammatory cells to sites of atherosclerosis development [92], (c) FAK, focal adhesion kinase, which is involved in integrin-dependent cell-to-matrix adhesion signaling, is important for migration in the extracellular matrix [93], (d) members of the JAK/STAT pathway, such as STAT5, are involved in SMCs activation in atherosclerosis [84] and (e) MMPs which are major extracellular proteolysis enzymatic systems that modify ECM, have evident roles during inflammatory and vascular diseases [94]. 

**Table 1:** Novel Findings of the Integrative Pathway Analysis of moxLDL-induced SMC Phenotype Transformation

**Theme**	**Novel Findings**
Cholesterol synthesis & transport	The observation of upregulated cholesterol biosynthesis coupled with a dysregulation of the reverse cholesterol transport pathway in moxLDL-induced SMC may promote the accumulation of cholesterol and cholesterol esters in the cells, and facilitate foam cell formation during atherogenesis
GPCR signaling	Observed GPCR signaling may have a role in SMC transformation. GPCR signaling following activation via inflammatory or other microenvironmental stimuli have been implicated in responses, such as change of cell-to-cell and cell-to-matrix adhesion, matrix remodeling, cell proliferation, migration, and immune cell trafficking and regulation. The activation of GPCR pathways may thus play a role in the initiation of SMC dedifferentiation and phenotype transformation.
Cytokine & growth factor	Potential role for IL12, IFN-α, HGF, CSF3 and VEGF signaling in SMC phenotype transformation.
Cell cycle control	Up-regulation of HBP1, a repressor of cyclin D1 and CDKN1B, suggests a negative feedback loop auto-triggered by the up-regulation of the core cell cycle machinery during SMC phenotype transformation.
Cell differentiation	The deregulation of the ID2 and ZEB1 regulators of differentiation during SMC phenotype transformation is consistent with the onset of SMC de-differentiation.
miRNA & de-differentiation	Potential role of miRNAs in the regulation of SMC phenotype transformation via interactions with the IFN-γ pathway.
Nexus genes in complex networks	Several “nexus” genes, including the components of a multi-subunit complex involved in the terminal stages of cholesterol synthesis, miRNAs (e.g. miR-203, miR-511, miR-590-3p, miR- 346*/miR-1207-5p/miR-4763-3p), members of the GPCR family of proteins (e.g. GPR1, GPR64, GPRC5A,GPR171, GPR176, GPR32, GPR25, and GPR124) and signal transduction pathways were observed. These genes may play important roles in VSMC phenotype transformation and in the pathogenesis of AT and coronary artery disease and may provide novel targets for drug discovery.

The original microarray data analyzed here has been deposited in the Gene Expression Omnibus database (accession GSE36487), and is publicly available for the first time with this paper.

Our enrichment map provides information regarding the activity status (up- or down-regulation) of GPCR signaling pathways during SMC transformation, while Ingenuity identifies cross-talk of this pathway with other pathways. Based on these observations, we speculate that GPCR signaling plays a role in SMC transformation. GPCR signaling may mediate the initiation of SMC dedifferentiation following activation via inflammatory or other microenvironmental stimuli. The activation of GPCR pathways might be implicated in a large number of responses, such as change of cell-to-cell/cell-to-matrix adhesion, proliferation, matrix remodeling, migration, and immune cell trafficking and regulation. These traits are consistent with the SMC transformation process. Once these processes have been completed, GPCR signaling is down-regulated, by a mechanism that is yet to be elucidated. The maintenance of the activated SMC phenotype could be regulated by other “maintenance pathways”, such as cytokine signaling pathways, which are up-regulated throughout the course of the disease. We believe that such maintenance pathways exist, given previous literature [22] and new evidence from our study that the migratory and proliferative phenotype in SMCs is maintained throughout moxLDL treatment by the strongly up-regulated cell cycle control machinery.

Members of the GPCR superfamily are known to mediate G-protein-coupled, cAMP-mediated signal transduction mechanisms for the detection of chemostimuli in the main olfactory epithelium and heterogeneous cells in mammals [95,96]. Since the olfactory sensing pathway was highly regulated in SMC exposed to moxLDL (Additional file 2: Figure S1), we speculate that in addition to moxLDL receptors, the GPCRs up-regulated in this process may participate in sensing this atherogenic agent.

#### Cell adhesion

**Figure 7:**
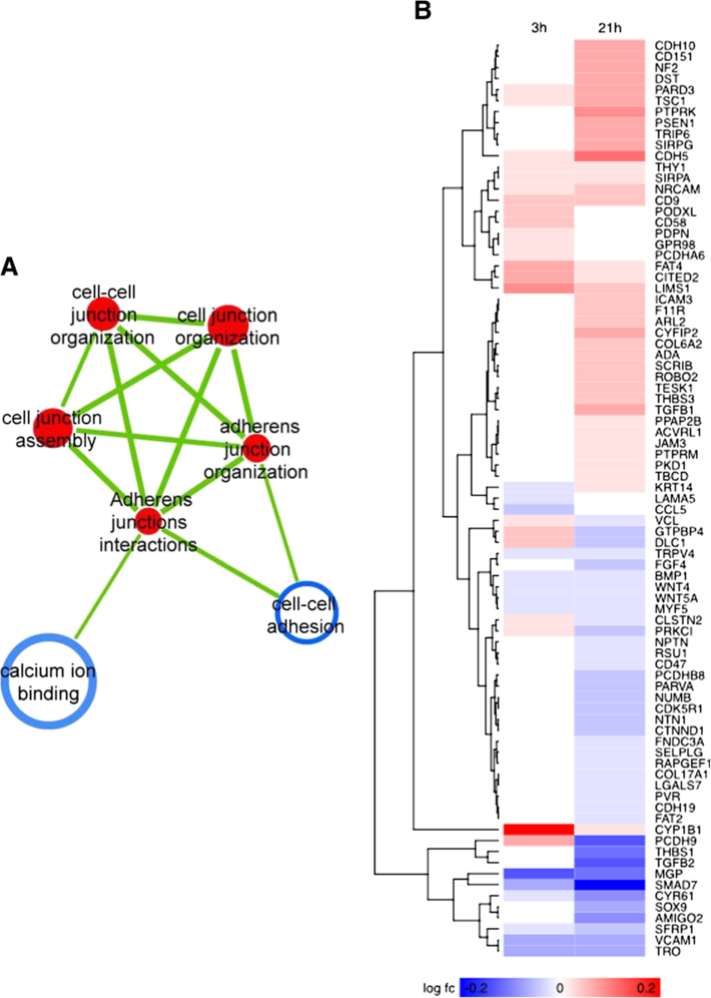
**Cell adhesion theme analysis.** (**A**) cell-to-cell junction theme from enrichment map. (**B**) Cell- to-cell junction-associated heatmap. For color coding interpretation, please refer to Figure 1.

SMC migration and proliferation induced by moxLDL contributes to the thickening of the intima in restenosis and AT. This process may be regulated by cadherins. Cadherins are transmembrane proteins which form cell-cell contacts. Studies by Uglow et al. (2003) [97] and Dwivedi et al. (2009) [98] have shown that MMP9 and -12-dependent shedding of the extracellular portion of N-cadherin results in the disruption of N-cadherin cell-cell contacts. This process was shown to be associated with the release and translocation of beta-catenin to the nucleus and the induction of beta-catenin-mediated intracellular signaling. This signaling cascade results in the expression of cyclin D1 and increased VSMC proliferation mediated by PDGF-BB [99,100]. These observations prompted us to analyze the cell-cell junction theme (Figure 7A). Significant alterations of the cell adhesion programming are implied via multiple ways in our analysis: (A) A cadherin expressional switch accompanies the SMC phenotypic transition; CDH10, CDH5 are up-regulated, while CDH19 and PCDH9 are down-regulated 21h post-moxLDL treatment (Figure 7B). Cadherins belong to the adherens junction apparatus, mediating cell-to-cell, homotypic cell adhesion coupling in epithelial or even stromal cells [101]. It has been postulated that molecular “switching” in these molecules contributes to the turnover of cell adhesion properties of the cells in various pathologies and participate in motile phenotypes, as in the case of cancer metastasis or even normal development [102-104]. (B) Cluster of differentiation (CD) molecule expressional switching, accompanies SMC transition; CD151 and CD9 are up-regulated, whereas CD47 is down-regulated with moxLDL treatment (Figure 7B). CD9 is a cell-surface glycoprotein belonging to the tetraspanin family of proteins, believed to be involved in complexes with integrins, thus mediating cell migration, adhesion and platelet aggregation [105]. CD151, belongs to the same family of proteins, additionally shown to accelerate cancer metastasis, thus promoting a migratory phenotype [106]. (C) Overexpression of PARD3 (Figure 7B), a member of the Par3/Par6 polarity complex, might suggest that SMC transition is accompanied by a polarized migration [107]. Taken together, these observations point to altered cell-adhesion machinery in the activated SMC that is consistent with a migratory phenotype. 

#### Cell cycle control

**Figure 8:**
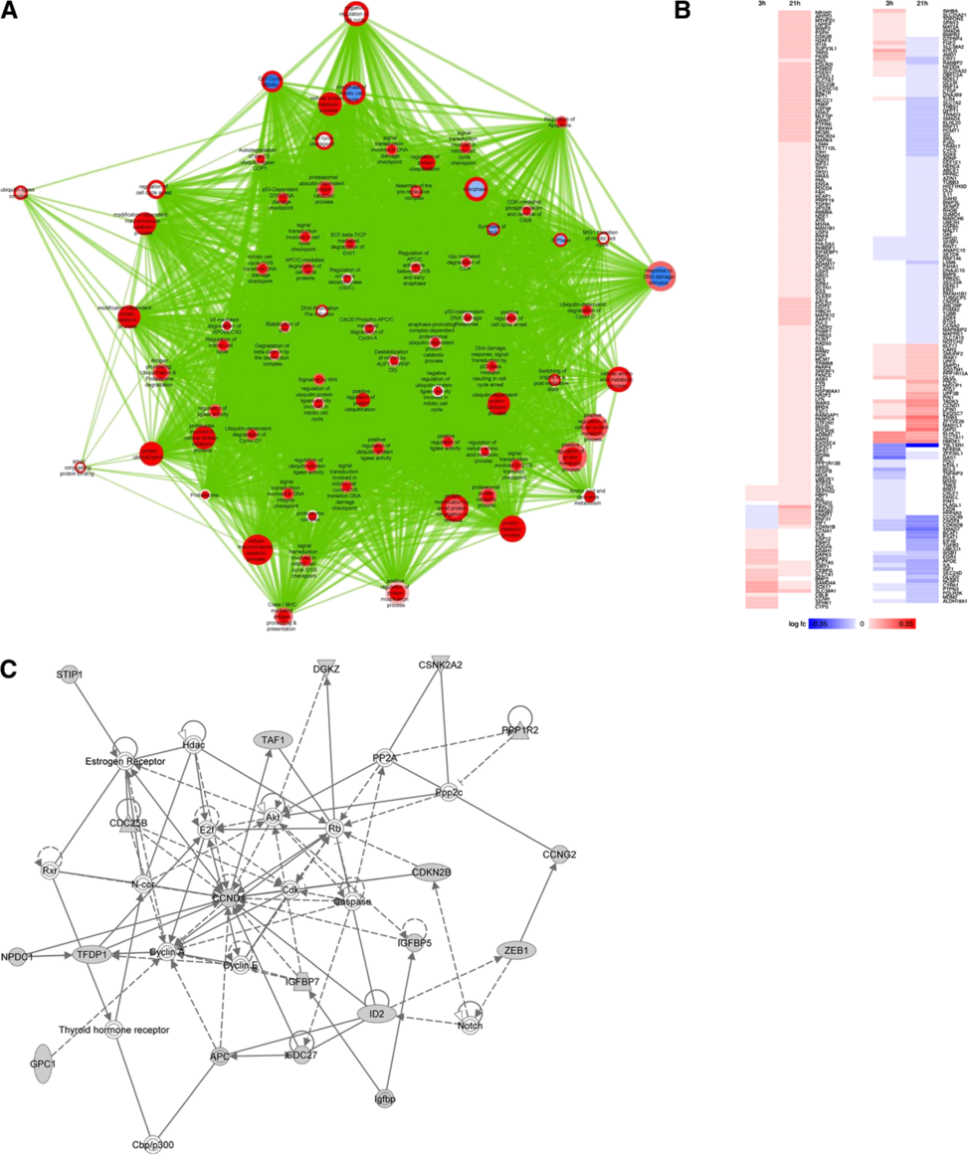
**Cell-cycle control theme analysis.** (**A**) Cell-cycle theme from enrichment map. (**B**) Cell-cycle- associated heatmap. (**C**) Cell-cycle-associated IPA network (21h). For color coding interpretation, please refer to Figures 1 &5.

The cell-cycle theme (Figure 8A) served as a proof-of-concept in our analysis, since cell cycle control and cell death machineries induce tremendous impact in tissue homeostasis of the adult organism, with known roles in inflammatory, vascular, neoplastic and neurodegenerative diseases [108]. Since the activated SMC phenotype is highly proliferative [4], disturbances in the cell-cycle control machinery are expected. The theme was clearly up-regulated in both time-points (Figure 8A, Table 1). Details drawn from the associated heatmap (Figure 8B) suggest that cyclin D1 is up-regulated and the cyclin-dependent kinase (CDKN) inhibitor, CDKN2B, is down-regulated, which is consistent with G1/S progression. Interestingly, HBP1 which is known to repress cyclin D1 [109], as well as CDKN1B are up-regulated, suggesting that a negative feedback loop to down-regulate cell growth could be already initiated, most probably triggered from the up-regulation of the core machinery. IPA also verified the involvement of the cell cycle control machinery in the 21h time point (Figure 8C). Specifically, proteins in our dataset are clustered around the key regulatory molecules of the cell cycle, such as the cyclin, the CDK, and the Rb/E2F family members (Figure 8C). 

#### Cell differentiation

An important aspect of SMC transition into a migratory and proliferative phenotype is the loss of the differentiated and quiescent phenotype. Regulatory factors of cell differentiation most likely regulate this transition. In one of our IPA networks (Figure 8C), we captured two potential regulators of differentiation: (a) DNA-binding protein inhibitor-2 (ID2), a transcriptional regulator which inhibits the function of basic helix-loop-helix transcription factors [110], and (b) Zinc-finger E-Box-binding homeobox 1 (ZEB1), a transcription factor involved in the generation of more mesenchymal phenotypes [29]. Interestingly, both ID2 and ZEB1 were deregulated in our dataset. Although IL-1β-induced ID2 gene expression has been described in SMCs [111], ZEB1 has not been reported to be involved in SMC phenotype transformation.

#### Myogenic contraction mechanism

**Figure 9:**
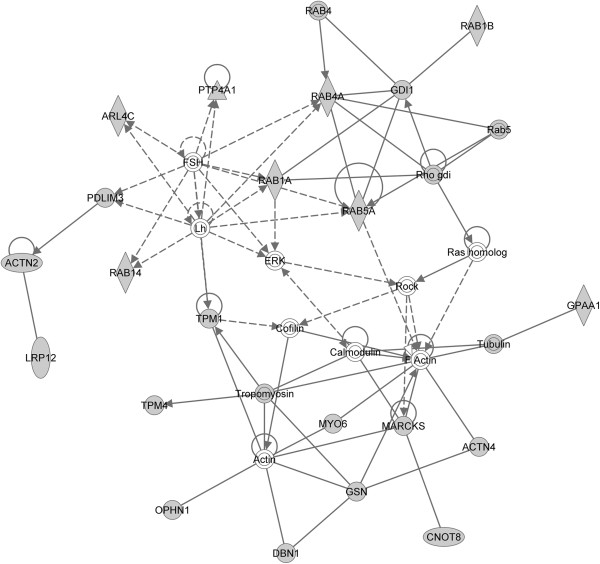
**Myogenic contraction-associated IPA network.** For color coding interpretation, please refer to Figure 5.

It has been reported that moxLDL induces a sustained and intense calcium-dependent retraction of SMC by down-regulation of the expression of genes responsible for the synthesis of SMC contractile proteins such as α-actin, smooth muscle myosin heavy chain-1, non-muscle myosin and calponin, a thin filament protein involved in the regulation of actin-myosin interactions [112]. moxLDL also stimulates collagen production, DNA synthesis and SMC proliferation [112,113]. A subnetwork of actin and actin-related gene/proteins was found in the 21h experiment (Figure 9). This network clusters molecules, such as myosin, tropomyosin and cofilin around actin filaments, involved in the myogenic contraction mechanism. Interestingly, the enrichment map reveals a large down-regulation of the theme “muscle function” in the 21h experiment (Figure 1). These observations are in concordance with cytoskeletal rearrangements, relevant to transformation of SMCs into the migratory phenotype [19]. 

** The novel findings in this paper are summarized in Table I.

## Conclusion

Pathway analysis of the transcriptomic data of the *in vitro* model of moxLDL induced-VSMC phenotype transformation using GSEA, Enrichment Map Cytoscape plugin, GeneMANIA and IPA software identified several pathways known or expected to be disturbed during SMC transformation in addition to several novel regulators that may play key roles in the onset and progression of AT. The identification of these novel potential regulatory genes now permit hypothesis generation and *in vivo* functional experimentation (such as gain and loss-of-function studies) to establish causality with the process of SMC phenotype transformation, AT and coronary artery disease and to possibly reveal novel diagnostic markers and targets for drug discovery.

## Abbreviations

ABCA: ATP-binding cassette (ABC) transporters A; ABCG: ATP-binding cassette (ABC) transporters G; ACAT1: Acetoacetyl CoA thiolase; AP-1: Adaptor protein-1; APOE: Apolipoprotein E; AT: Atherosclerosis; CCL19: CCL20, chemokines; CD: Cluster of differentiation; CDH: Cadherin; CDKN1B: Cyclin-dependent kinase inhibitor B; CSF: Colony stimulating factor; CXCL: Chemokine CXC motif, ligand; EGF: Epidermal growth factor; FGF: Fibroblast growth factor; FAK: Focal adhesion kinase; GPCR: GTP-binding protein (G-protein)-coupled receptor; GPR: G-protein coupled receptor; GSEA: Gene Set Enrichment Analysis; HBP1: High density lipoprotein-binding protein 1; HGF: Hepatic growth factor; HMGCS1: 3-hydroxy-3-methylglutaryl-CoA synthetase 1; HMGCR: HMG-CoA reductase; ID2: Inhibitor of DNA binding-2; IDI1: Isopentenyl-diphosphate delta-isomerase 1; IFNγ: Interferon γ; IGF: Insulin-like growth factor; IL: Interleukin; IL1α: Interleukin 1α; INSIG1: Insulin-induced gene 1; INSIG2: Insulin-induced gene 2; IPA: Ingenuity pathway analysis; IPKB: Ingenuity pathways knowledge base; IRF: Interferon regulatory factor; LDLR: Low density lipoprotein receptor; PKC: Protein kinase-C; MAPK: Mitogen-activated protein kinase; MCP-1: Monocyte chemotactic protein-1; MD-2: Lymphocyte antigen 96; miR: MicroRNA; MMP: Matrix metalloproteinase; moxLDL: Minimally oxidized LDL; NFkB: Nuclear factor kappa B; NSDHL: NAD(P)H steroid dehydrogenase-like protein; PARD3: Partitioning-defective protein 3; PCDH: Protocadherin; PDGF: Platelet-derived growth factor; PI3K: Phosphatidylinositol 3-kinase; SCAP: SREBP cleavage-activating protein; SMCs: Smooth muscle cells; SREBP: Sterol regulatory element-binding protein; STAT: Signal transducer and activator of transcription; TGF-β: Transforming growth factor beta; TLR4: Toll-like receptor 4; VEGF: Vascular endothelial cell growth factor; (V)LDL: (very) low density lipoprotein; VSMC: Vascular smooth muscle cell; ZEB1: Zinc-finger E-Box-binding homeobox 1.

## Competing interests

The authors declare no competing interests.

## Authors’ contributions

The project was conceived by JM. Experiments and pathway analyses were performed by GSK, JW, GDB and JM. The obtained results were interpreted by GSK, GDB, JW and JM. The manuscript was written and approved by all authors.

## Pre-publication history

The pre-publication history for this paper can be accessed here:

http://www.biomedcentral.com/1471-2261/13/4/prepub

## Supplementary Material

Additional file 1 **Table S1.** List of functional themes (Column A), taken from the enrichment map (Figure 1), with their explained behavior, according to the node colors, at 3h (column B) and 21h (Column C) time points. Click here for file

Additional file 2 **Figure S1.** Heat maps of SMC differential gene expression patterns, induced by treatment with moxLDL at 3h and 21h. Partial information from these heat maps was used to draw Figures 4, 6, 7 and 8. Click here for file

Additional file 3 **Table S2.** IPA results. All the top-listed canonical (metabolic & signaling) pathways using the Fisher’s exact test in the 3h (Additional file 1: Table S1) and 21h (Additional file 2: Table S2) moxLDL experiment, respectively. Column A depicts canonical pathway name as tagged in the Ingenuity knowledgebase; column B depicts the Fisher exact test –log(P-Value); column C depicts the statistical threshold; column D provides the correspondence of the moxLDL differentially expressed genes to each of the individual canonical pathways. Partial information from these tables was plotted as Figures 3A and 3B in this manuscript. Click here for file

Additional file 4 **Table S3.** IPA results. All the top-listed canonical (metabolic & signaling) pathways using the Fisher’s exact test in the 3h (Additional file 1: Table S1) and 21h (Additional file 2: Table S2) moxLDL experiment, respectively. Column A depicts canonical pathway name as tagged in the Ingenuity knowledgebase; column B depicts the Fisher exact test –log(P-Value); column C depicts the statistical threshold; column D provides the correspondence of the moxLDL differentially expressed genes to each of the individual canonical pathways. Partial information from these tables was plotted as Figures 3A and 3B in this manuscript. Click here for file
