# Supplementary material for: Integrative pathway dissection of molecular mechanisms of moxLDL-induced vascular smooth muscle phenotype transformation
Source: BMC Cardiovasc Disord. 2013 Jan 16;13:4. doi: 10.1186/1471-2261-13-4 (PMC3556327; doi:10.1186/1471-2261-13-4)

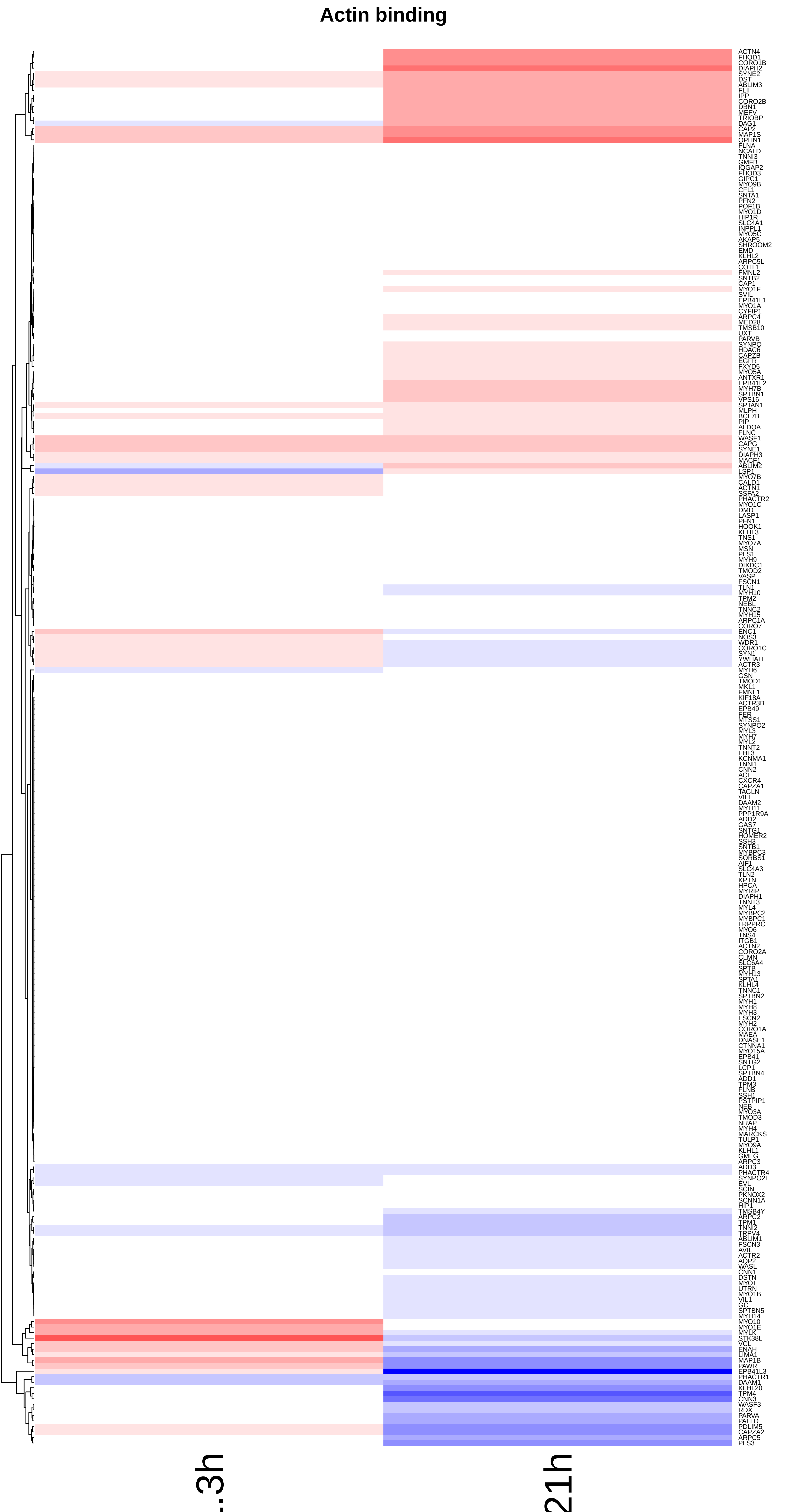
Actin binding
ACTN4
FHOD1
CORO1B
DIAPH2
SYNE2
DST
ABLIM3
FLII
IPP
CORO2B
DBN1
MEFV
TRIOBP
DAG1
CAP2
MAP1S
OPHN1
FLNA
NCALD
TNNI3
GMFB
IQGAP2
FHOD3
GIPC1
MYO9B
CFL1
SNTA1
PFN2
POF1B
MYO1D
HIP1R
SLC4A1
INPPL1
MYO5C
AKAP5
SHROOM2
EMD
KLHL2
ARPC5L
COTL1
FMNL2
SNTB2
CAP1
MYO1F
SVIL
EPB41L1
MYO1A
CYFIP1
ARPC4
MED28
TMSB10
UXT
PARVB
SYNPO
HDAC6
CAPZB
EGFR
FXYD5
MYO5A
ANTXR1
EPB41L2
MYH7B
SPTBN1
VPS16
SPTAN1
MLPH
BCL7B
PIP
ALDOA
FLNC
WASF1
CAPG
SYNE1
DIAPH3
MACF1
ABLIM2
LSP1
MYO7B
CALD1
ACTN1
SSFA2
PHACTR2
MYO1C
DMD
LASP1
PFN1
HOOK1
KLHL3
TNS1
MYO7A
MSN
PLS1
MYH9
DIXDC1
TMOD2
VASP
FSCN1
TLN1
MYH10
TPM2
NEBL
TNNC2
MYH15
ARPC1A
CORO7
ENC1
NOS3
WDR1
CORO1C
SYN1
YWHAH
ACTR3
MYH6
GSN
TMOD1
MKL1
FMNL1
KIF18A
ACTR3B
EPB49
FER
MTSS1
SYNPO2
MYL3
MYH7
MYL2
TNNT2
FHL3
KCNMA1
TNNI1
CNN2
ACE
CXCR4
CAPZA1
TAGLN
VILL
DAAM2
MYH11
PPP1R9A
ADD2
GAS7
SNTG1
HOMER2
SSH3
SNTB1
MYBPC3
SORBS1
AIF1
SLC4A3
TLN2
KPTN
HPCA
MYRIP
DIAPH1
TNNT3
MYL4
MYBPC2
MYBPC1
LRPPRC
MYO6
TNS4
ITGB1
ACTN2
CORO2A
CLMN
SLC6A4
SPTB
MYH13
SPTA1
KLHL4
TNNC1
SPTBN2
MYH1
MYH8
MYH3
FSCN2
MYH2
CORO1A
MAEA
DNASE1
CTNNA1
MYO15A
EPB41
SNTG2
LCP1
SPTBN4
ADD1
TPM3
FLNB
SSH1
PSTPIP1
NEB
MYO3A
TMOD3
NRAP
MYH4
MARCKS
TULP1
MYO9A
KLHL1
GMFG
ARPC3
ADD3
PHACTR4
SYNPO2L
EVL
SCIN
PKNOX2
SCNN1A
HIP1
TMSB4Y
ARPC2
TPM1
TNNI2
TRPV4
ABLIM1
FSCN3
AVIL
ACTR2
AQP2
WASL
CNN1
DSTN
MYOT
UTRN
MYO1B
VIL1
GC
SPTBN5
MYH14
MYO10
MYO1E
MYLK
STK38L
VCL
ENAH
LIMA1
MAP1B
PAWR
EPB41L3
PHACTR1
DAAM1
KLHL20
TPM4
CNN3
WASF3
RDX
PARVA
PALLD
PDLIM5
CAPZA2
ARPC5
PLS3
3h
21h

Cation transporters

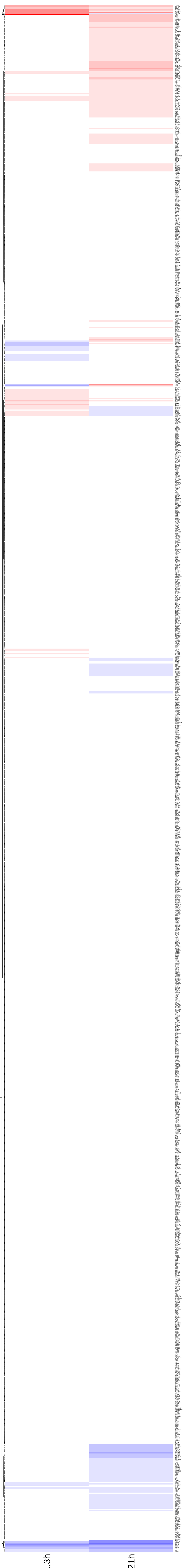

Cell cycle

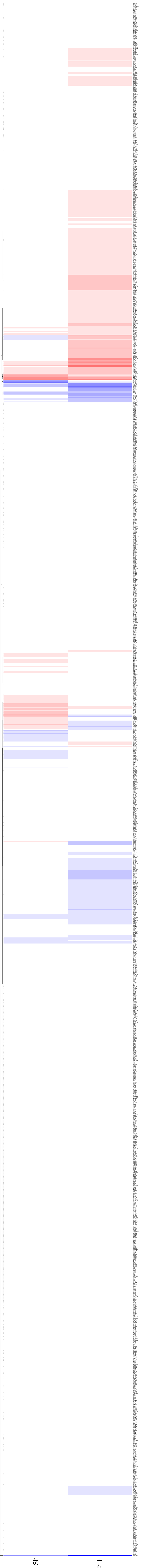

Chromatin Organization

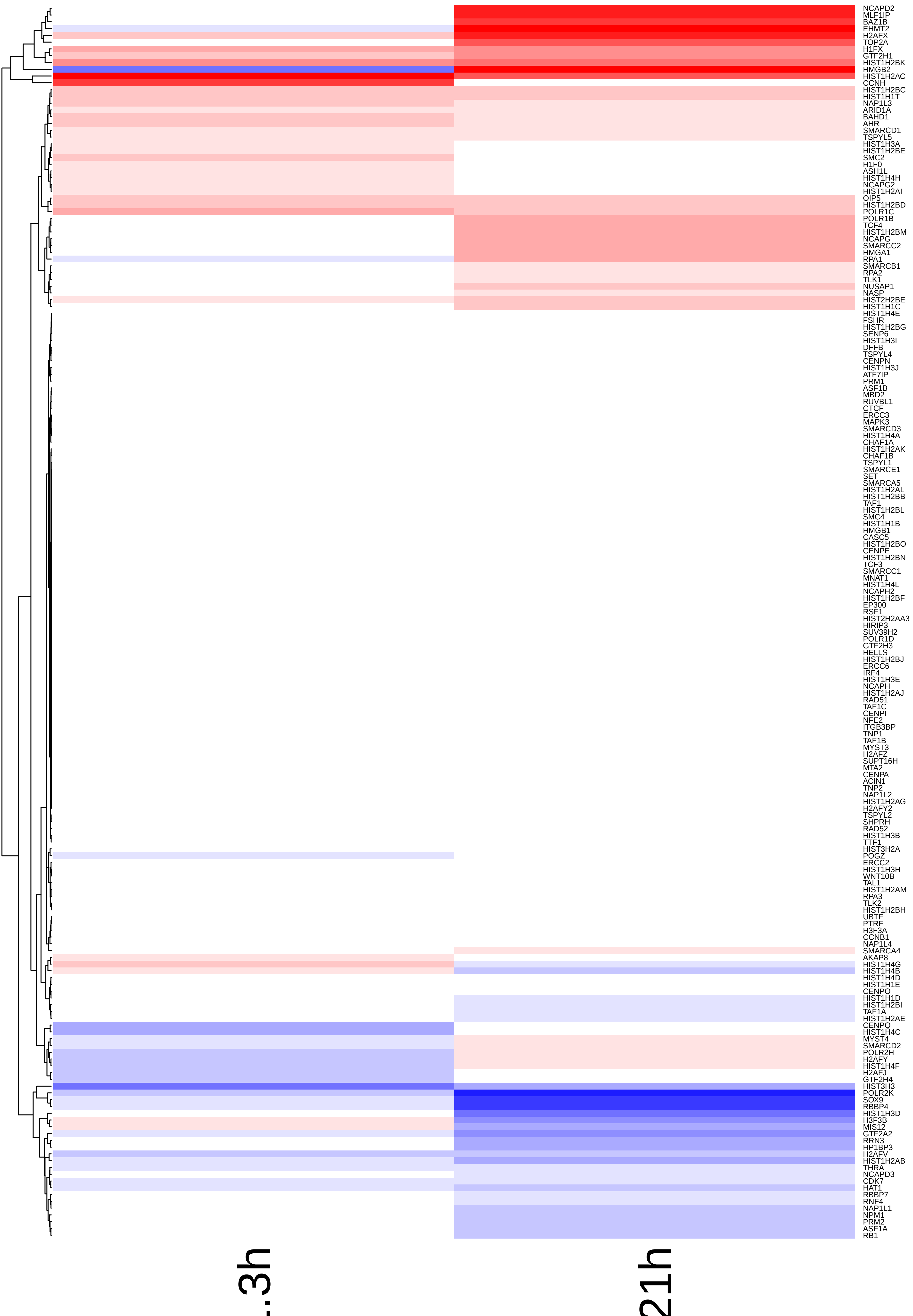

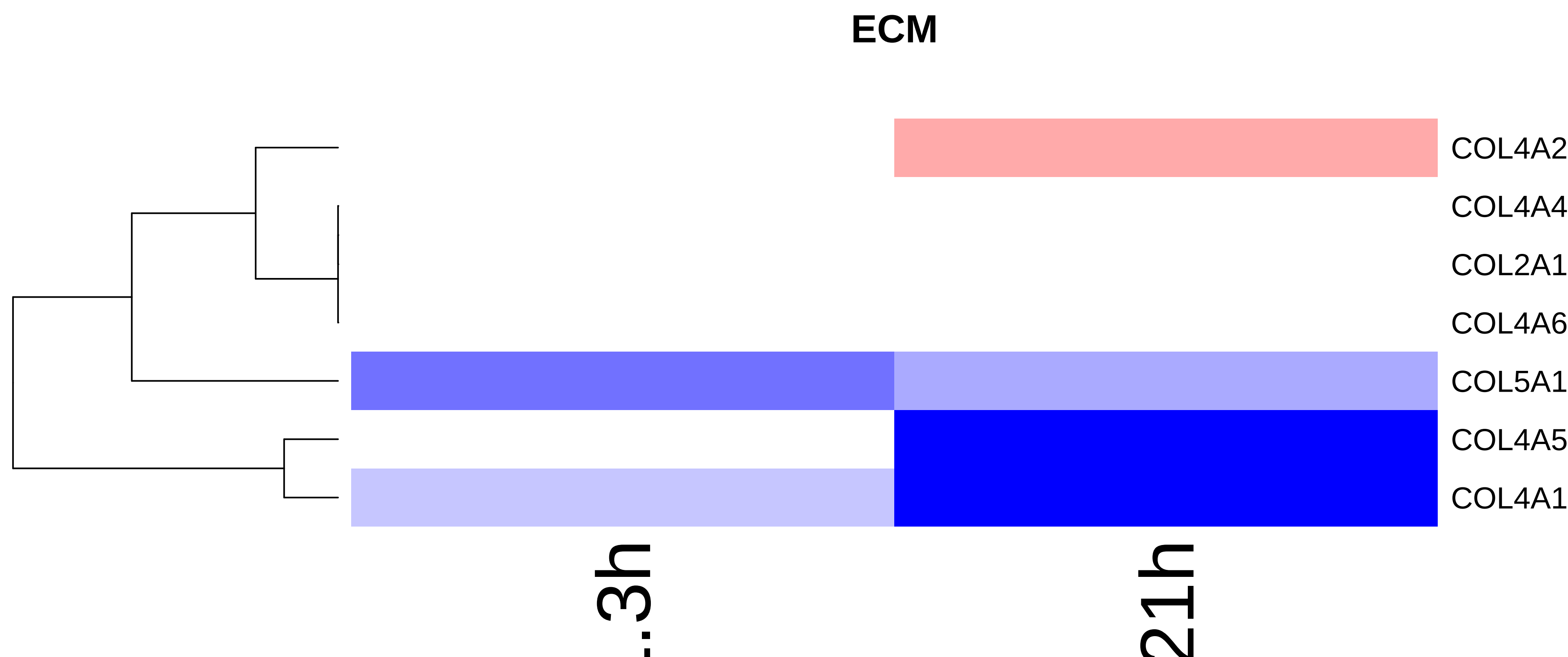
ECM
COL4A2
COL4A4
COL2A1
COL4A6
COL5A1
COL4A5
COL4A1
3h
21h

EGFR1

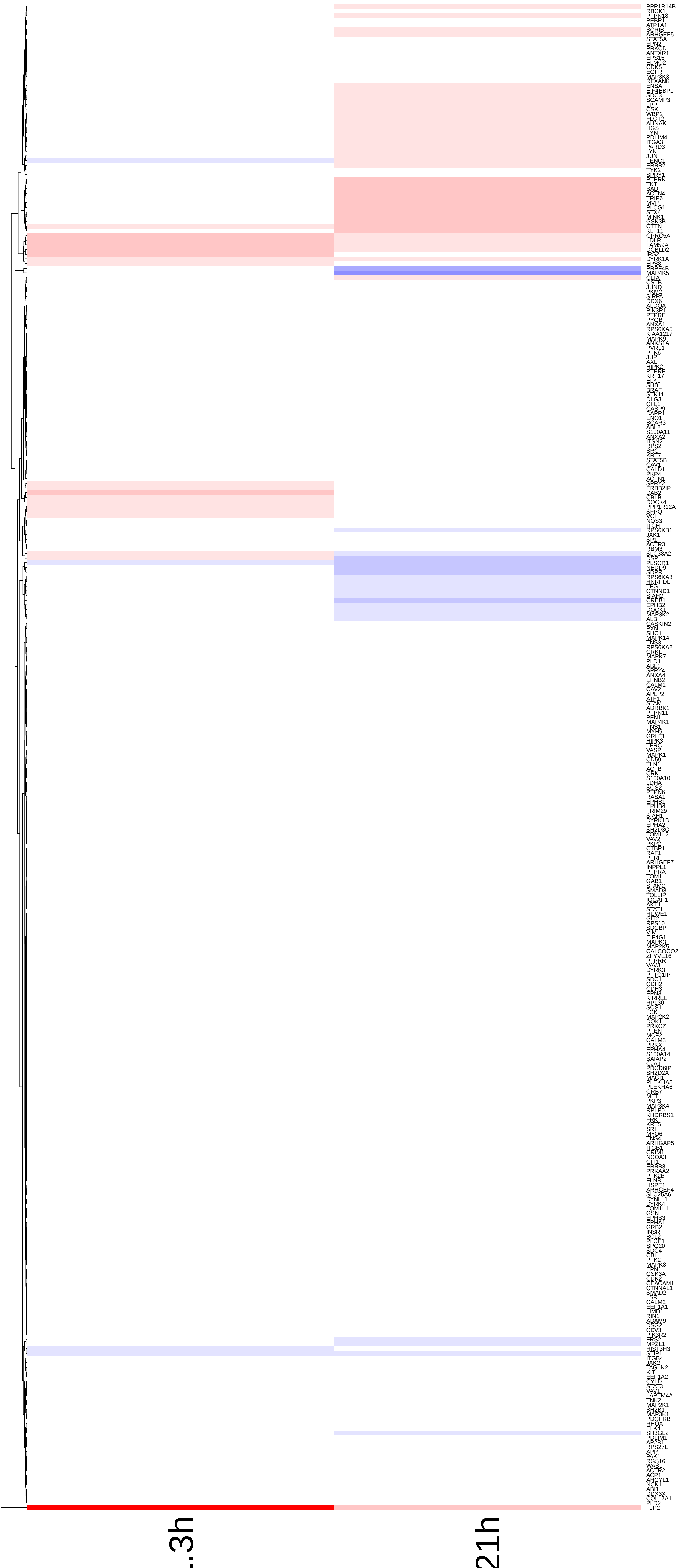
PPP1R14B
RBCK1
PTPN18
PEBP1
ATP1A1
SCRIB
ARHGEF5
STAT5A
EPN2
PRKCD
ANTXR1
EPS15
ELMO2
CDK5
EGFR
MAP3K3
RFXANK
ENSA
EIF4EBP1
SDC3
SCAMP3
LPP
CSK
WBP2
FLOT2
AHNAK
HGS
FYN
PDLIM4
ITGA3
PARD3
LYN
JUN
TENC1
ERBB2
TYK2
SPRY1
PTPRK
TKT
BAD
ACTN4
TRIP6
MVP
PLCG1
STX4
MINK1
GSK3B
CTTN
KLF11
GPRC5A
LDLR
FAM59A
DCBLD2
IRS2
DYRK1A
EPS8
PRPF4B
MAP4K5
CLTA
CSTB
JUND
PKM2
SIRPA
DDX6
ALDOA
PIK3R1
PTPRE
PYGB
ANXA1
RPS6KA5
KIAA1217
MAPK9
ANKS1A
PVRL1
PTK6
JUP
AXL
HIPK2
PTPRF
KRT17
ELK1
SHB
BRAF
STK11
DLG3
CFL1
CASP9
DAPP1
ENO1
BCAR3
ABL2
S100A11
ANXA2
ITSN2
RPS2
SRC
KRT7
STAT5B
CAV1
CALD1
PKP4
ACTN1
SPRY2
ERBB2IP
DAB2
CBLB
DOCK4
PPP1R12A
SFPQ
VCL
NOS3
ITCH
RPS6KB1
JAK1
SP1
ACTR3
RBM3
SLC38A2
DSP
PLSCR1
NEDD9
SDPR
RPS6KA3
HNRPDL
TFG
CTNND1
SIAH2
CREB1
EPHB2
DOCK1
MAP3K2
ALB
CASKIN2
PXN
SHC1
MAPK14
TNS3
RPS6KA2
CRKL
MAPK7
PLD1
ABL1
SPRY4
ANXA4
EFNB2
CALM1
CAV2
APLP2
ATF1
STAM
ADRBK1
PTPN11
PFN1
MAP4K1
TNS1
MYH9
GRLF1
HIPK3
TFRC
VASP
MAPK1
CD59
TLN1
ACTB
CRK
S100A10
LDHA
SOS2
PTPN6
RASA1
EPHB1
EPHB4
TRIM29
SIAH1
DYRK1B
EPHA2
SH2D3C
TOM1L2
VAV2
PKP2
CTBP1
RAF1
PTRF
ARHGEF7
INPPL1
PTPRA
TOM1
GAB1
STAM2
SMAD3
TOLLIP
IQGAP1
AKT1
STAT1
HUWE1
GIT2
RPS10
SDCBP
VIM
EIF4G1
MAPK3
MAP2K5
CALCOCO2
ZFYVE16
PTPRR
VAV3
DYRK3
PTTG1IP
SDC1
CDH2
CDH3
EPN3
KIRREL
RPL30
SOS1
LCK
MAP2K2
DOK1
PRKCZ
PTEN
MCF2
CALM3
PRKX
EPHA4
S100A14
BAIAP2
GJA1
PDCD6IP
SH2D2A
MAGI1
PLEKHA5
PLEKHA6
GRB7
MET
PKP3
MAP3K4
RPLP0
KHDRBS1
FRK
KRT5
SRI
MYO6
TNS4
ARHGAP5
ITGB1
CRIM1
NCOA3
GIT1
ERBB3
PRKAA2
PTK2B
FLNB
HSPE1
ARHGEF4
SLC25A6
DYNLL1
DYRK4
TOM1L1
GSN
EPHB3
EPHA1
GRB2
INSR
BCL2
PLCE1
SPG20
SDC4
CBL
PTK2
MAPK8
EPN1
GSK3A
CDK2
CEACAM1
CTNNAL1
SMAD2
LSR
CALM2
EEF1A1
LIMD1
RIN1
ADAM9
DSG2
CDV3
PIK3R2
FRS2
MPZL1
HIST3H3
STIP1
ITGB4
JAK2
TAGLN2
KIT
EEF1A2
CYLD
STAT3
VAV1
LAPTM4A
TNK2
MAP2K1
SH2B1
MAP3K1
PDGFRB
RHOA
ELK4
SH3GL2
PDLIM1
AP2B1
RPS27L
APP
PAK1
RGS16
WASL
ACTR2
ACP1
AHCYL1
NCK1
ABI1
DDX3X
COL17A1
PLD2
TJP2
3h
21h

Endopeptidase

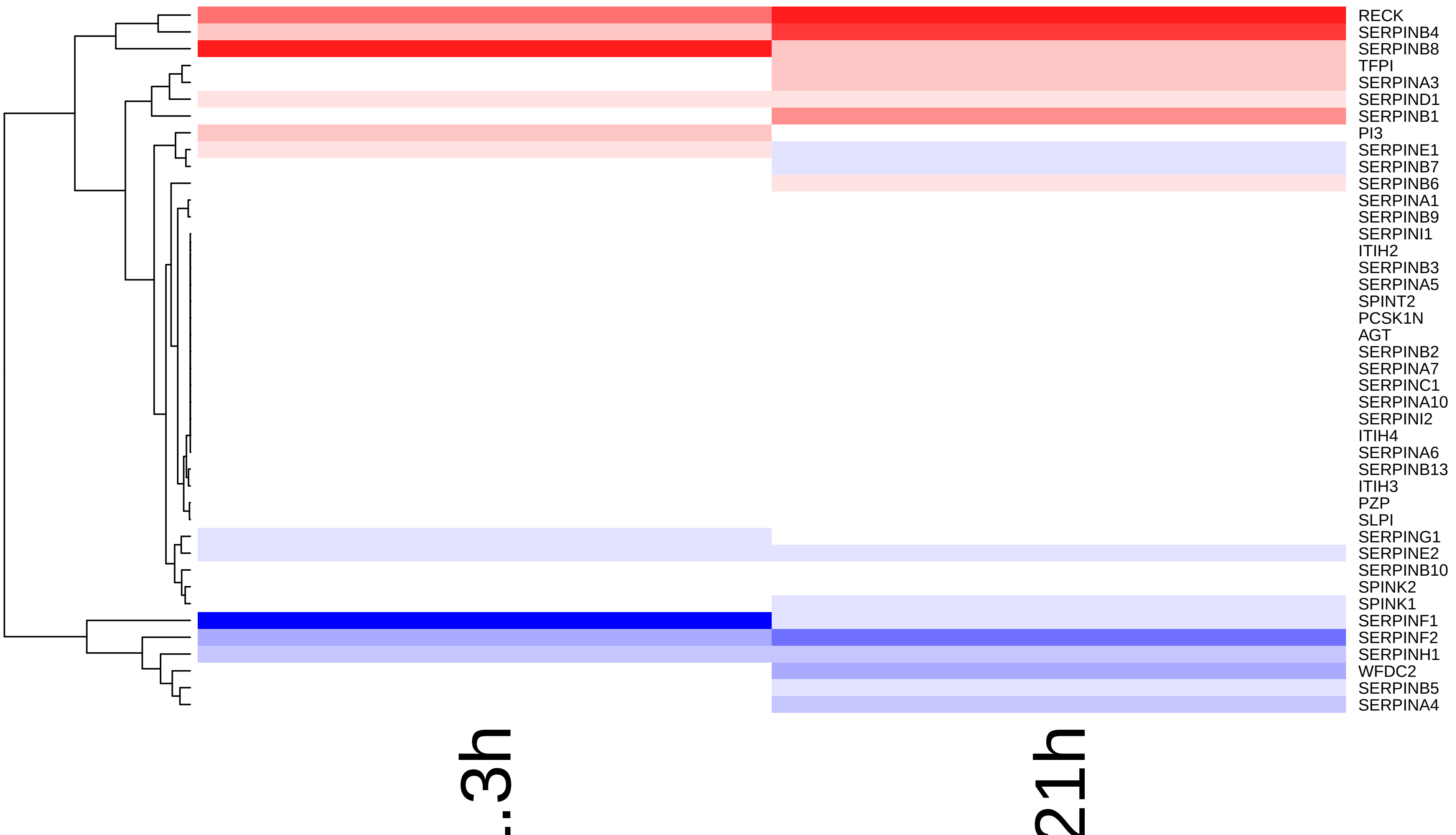

GPCR Signaling

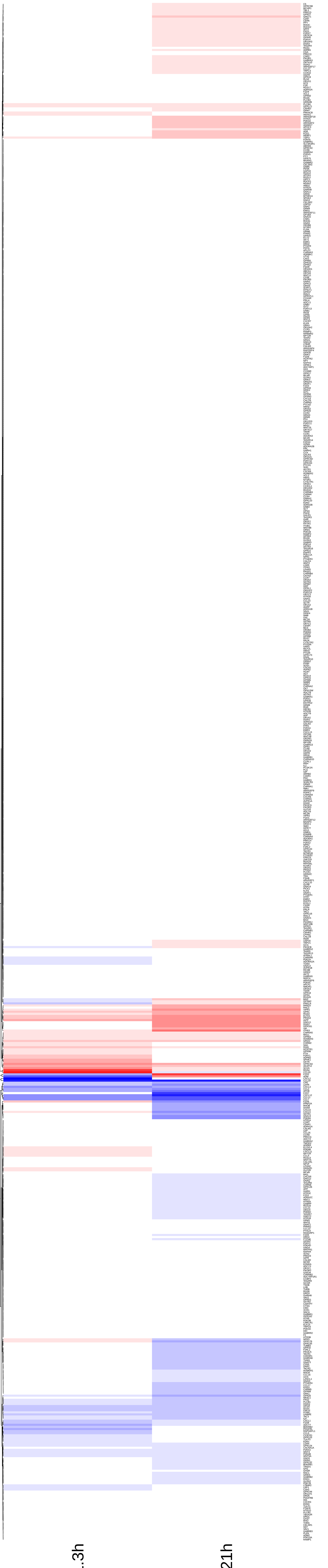

Lysosome

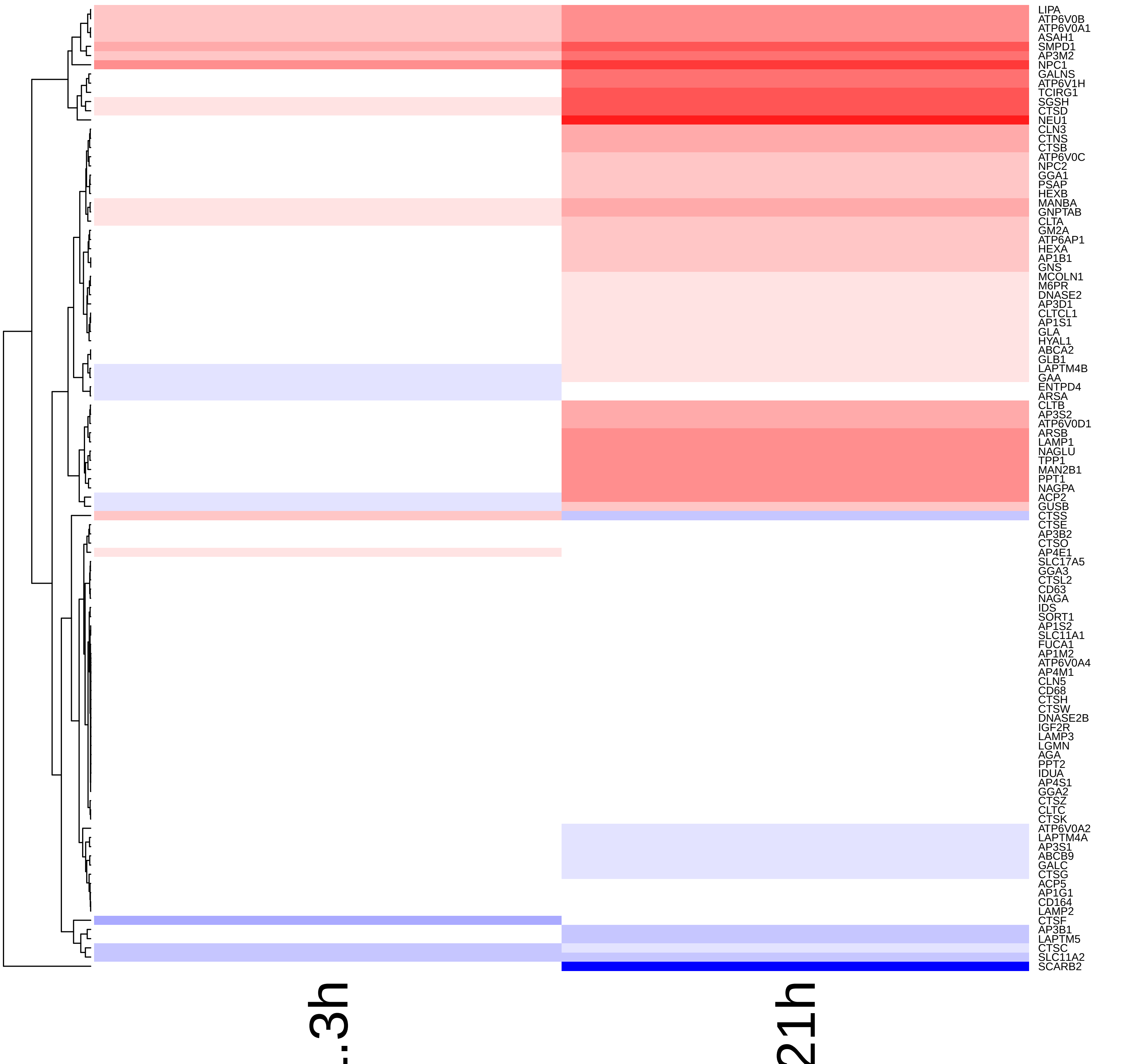

LIPA
ATP6V0B
ATP6V0A1
ASAH1
SMPD1
AP3M2
NPC1
GALNS
ATP6V1H
TCIRG1
SGSH
CTSD
NEU1
CLN3
CTNS
CTSB
ATP6V0C
NPC2
GGA1
PSAP
HEXB
MANBA
GNPTAB
CLTA
GM2A
ATP6AP1
HEXA
AP1B1
GNS
MCOLN1
M6PR
DNASE2
AP3D1
CLTCL1
AP1S1
GLA
HYAL1
ABCA2
GLB1
LAPTM4B
GAA
ENTPD4
ARSA
CLTB
AP3S2
ATP6V0D1
ARSB
LAMP1
NAGLU
TPP1
MAN2B1
PPT1
NAGPA
ACP2
GUSB
CTSS
CTSE
AP3B2
CTSO
AP4E1
SLC17A5
GGA3
CTSL2
CD63
NAGA
IDS
SORT1
AP1S2
SLC11A1
FUCA1
AP1M2
ATP6V0A4
AP4M1
CLN5
CD68
CTSH
CTSW
DNASE2B
IGF2R
LAMP3
LGMN
AGA
PPT2
IDUA
AP4S1
GGA2
CTSZ
CLTC
CTSK
ATP6V0A2
LAPTM4A
AP3S1
ABCB9
GALC
CTSG
ACP5
AP1G1
CD164
LAMP2
CTSF
AP3B1
LAPTM5
CTSC
SLC11A2
SCARB2
3h
21h

Muscle function

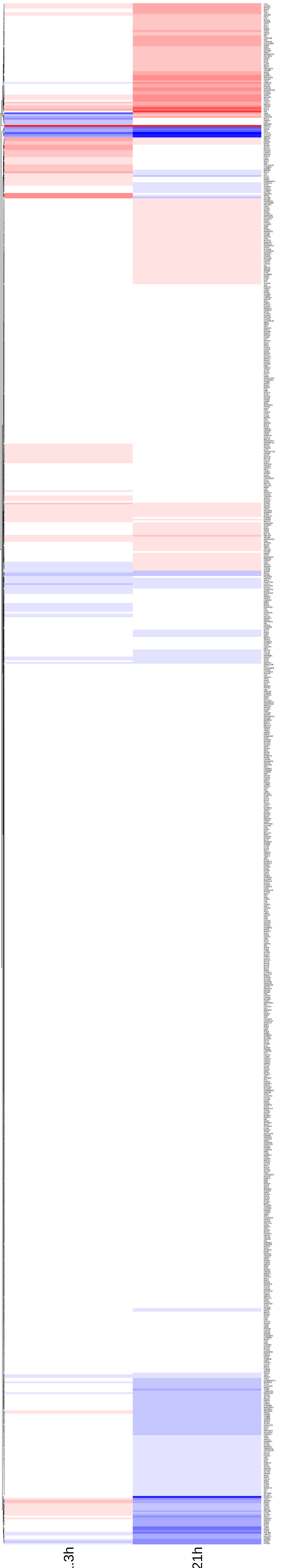

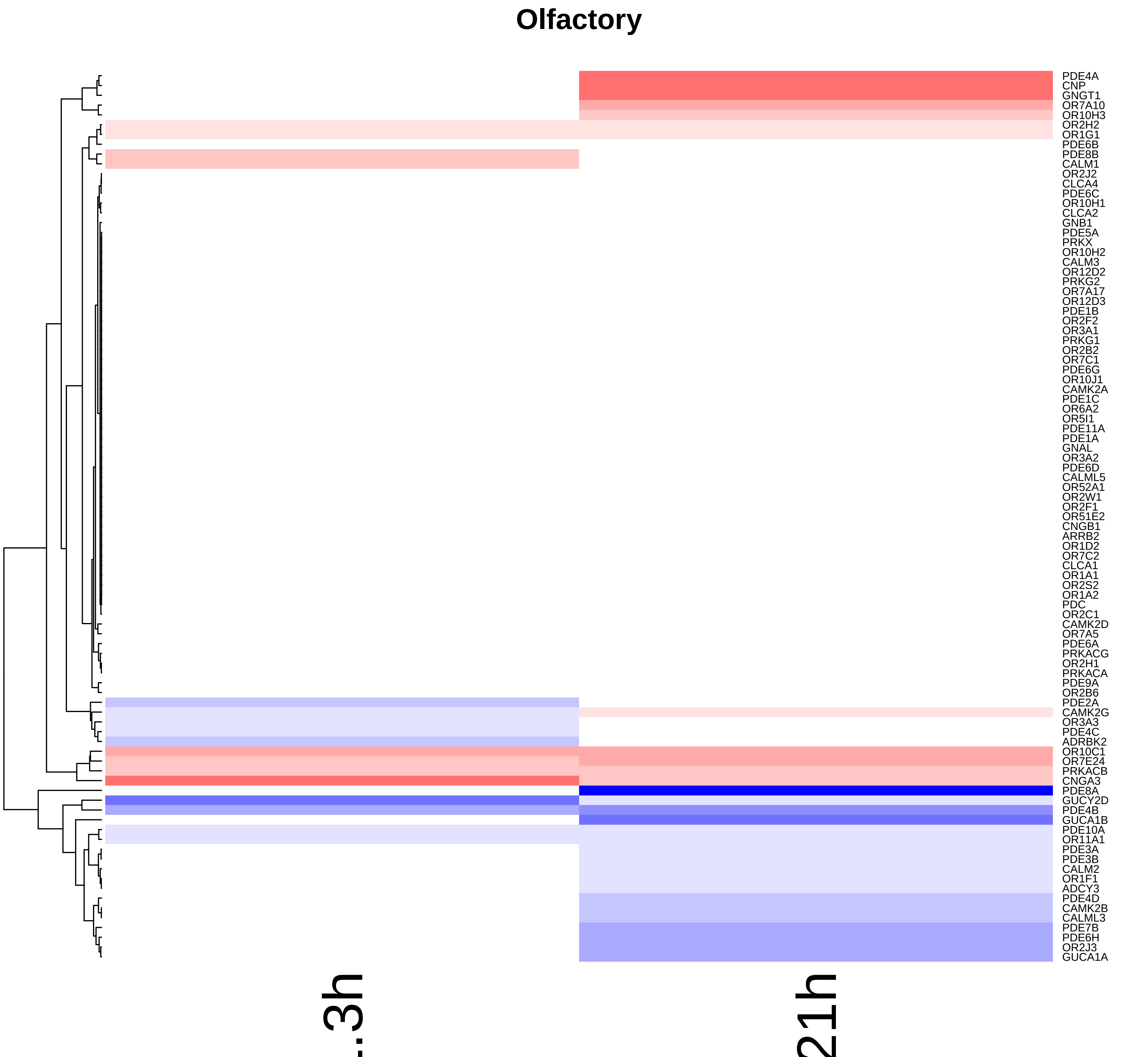

Olfactory
PDE4A
CNP
GNGT1
OR7A10
OR10H3
OR2H2
OR1G1
PDE6B
PDE8B
CALM1
OR2J2
CLCA4
PDE6C
OR10H1
CLCA2
GNB1
PDE5A
PRKX
OR10H2
CALM3
OR12D2
PRKG2
OR7A17
OR12D3
PDE1B
OR2F2
OR3A1
PRKG1
OR2B2
OR7C1
PDE6G
OR10J1
CAMK2A
PDE1C
OR6A2
OR5I1
PDE11A
PDE1A
GNAL
OR3A2
PDE6D
CALML5
OR52A1
OR2W1
OR2F1
OR51E2
CNGB1
ARRB2
OR1D2
OR7C2
CLCA1
OR1A1
OR2S2
OR1A2
PDC
OR2C1
CAMK2D
OR7A5
PDE6A
PRKACG
OR2H1
PRKACA
PDE9A
OR2B6
PDE2A
CAMK2G
OR3A3
PDE4C
ADRBK2
OR10C1
OR7E24
PRKACB
CNGA3
PDE8A
GUCY2D
PDE4B
GUCA1B
PDE10A
OR11A1
PDE3A
PDE3B
CALM2
OR1F1
ADCY3
PDE4D
CAMK2B
CALML3
PDE7B
PDE6H
OR2J3
GUCA1A
21h

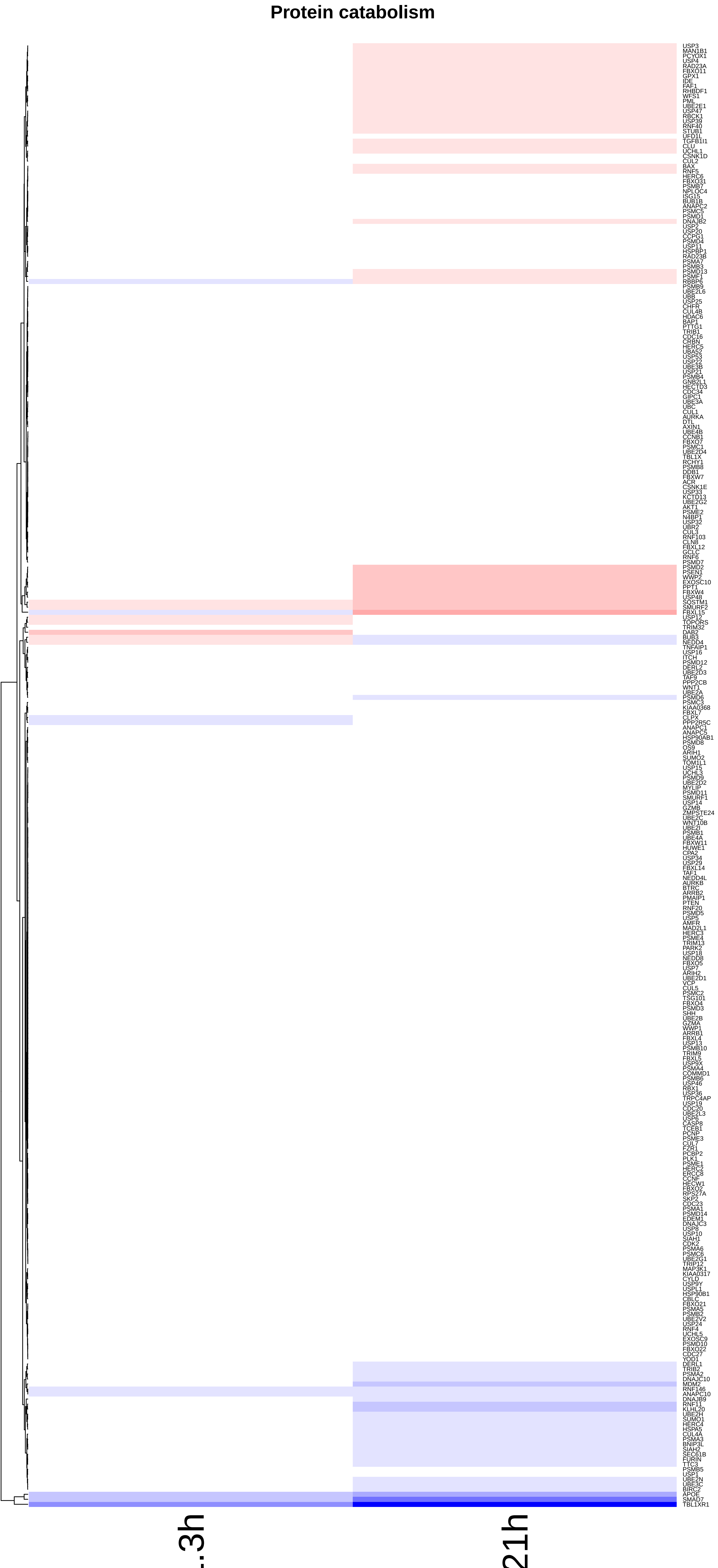
Protein catabolism
USP3
MAN1B1
PCYOX1
USP4
RAD23A
FBXO11
GPX1
IDE
FAF1
RHBDF1
WFS1
PML
UBE2E1
USP47
RBCK1
USP39
RNF40
STUB1
UFD1L
TGFB1I1
CLU
UCHL1
CSNK1D
CUL2
BAX
RNF5
HERC6
FBXO31
PSMB7
NPLOC4
ISG15
BUB1B
ANAPC2
PSMC5
PSMD1
DNAJB2
USP2
USP20
CCPG1
PSMD4
USP11
HSPBP1
RAD23B
PSMA7
PSMB3
PSMD13
PSMF1
RBBP6
PSMB9
UBE2L6
UBB
USP25
CHFR
CUL4B
HDAC6
BAP1
PTTG1
TRIB1
CDC16
CRBN
HERC5
UBA52
USP53
USP22
UBE3B
USP21
PSMB4
GNB2L1
HECTD3
CDC34
GIPC1
UBE3A
UBC
CUL1
AURKA
DTL
AXIN1
UBE4B
CCNB1
FBXO7
PSMC1
UBE2D4
TBL1X
RCHY1
PSMB8
DDB1
FBXW7
ACR
CSNK1E
USP33
KCTD13
UBE2G2
AKT1
PSME2
N4BP1
USP32
UBR2
CUL3
RNF103
CLN8
FBXL12
GCLC
RNF6
PSMD7
PSMD2
PSEN1
WWP2
EXOSC10
PPT1
FBXW4
USP48
SQSTM1
SMURF2
FBXL15
USP12
TOPORS
TRIM32
DAB2
BUB3
NEDD4
TNFAIP1
USP16
ITCH
PSMD12
DERL2
UBE2D3
TAF9
PPP2CB
WNT1
UBE2A
PSMD6
PSMC3
KIAA0368
FBXL7
CLPX
PPP2R5C
ANAPC1
ANAPC5
HSP90AB1
PSMD8
OS9
ARIH1
SUMO2
TOM1L1
USP15
UCHL3
PSMD9
UBE2D2
MYLIP
PSMD11
SMURF1
USP14
GZMB
ZMPSTE24
UBE2C
WNT10B
UBE2I
PSMB1
UBE4A
FBXW11
HUWE1
CPA2
USP34
USP29
FBXL14
TAF1
NEDD4L
AURKB
BTRC
ARRB2
PMAIP1
PTEN
RNF20
PSMD5
USP5
AMFR
MAD2L1
HERC3
PSME4
TRIM13
PARK2
USP18
NEDD8
FBXO5
USP7
ARIH2
UBE2D1
VCP
CUL5
PSMC2
TSG101
FBXO4
PSMD3
SHH
UBE2B
GZMA
WWP1
ARRB1
FBXL4
USP13
PSMB10
TRIM9
FBXL5
USP9X
PSMA4
COMMD1
PSMB6
USP46
RBX1
USP36
TRPC4AP
USP19
CDC20
UBE2L3
USP6
CASP8
TCEB1
PCNP
PSME3
CUL7
FZR1
PCBP2
PLK1
PSME1
HERC2
ERCC8
CCNF
HECW1
FBXO2
RPS27A
SKP2
CDC23
PSMA1
PSMD14
EDEM1
DNAJC3
USP8
USP10
SIAH1
CDK2
PSMA6
PSMC6
UBE2G1
TRIP12
MAP3K1
KIAA0317
CYLD
USP9Y
USPL1
HSP90B1
CBLC
FBXO21
PSMA5
PSMB2
UBE2V2
USP24
RNF4
UCHL5
EXOSC9
PSMD10
FBXO22
CDC27
YOD1
DERL1
TRIB2
PSMA2
DNAJC10
MDM2
RNF146
ANAPC10
DNAJB9
RNF11
KLHL20
UBE2H
SUMO1
HERC4
HSPA5
CUL4A
PSMA3
BNIP3L
SIAH2
SEC61B
FURIN
TTC3
PSMB5
USP1
UBE2N
UBE3C
BIRC2
APOE
SMAD7
TBL1XR1
3h
21h

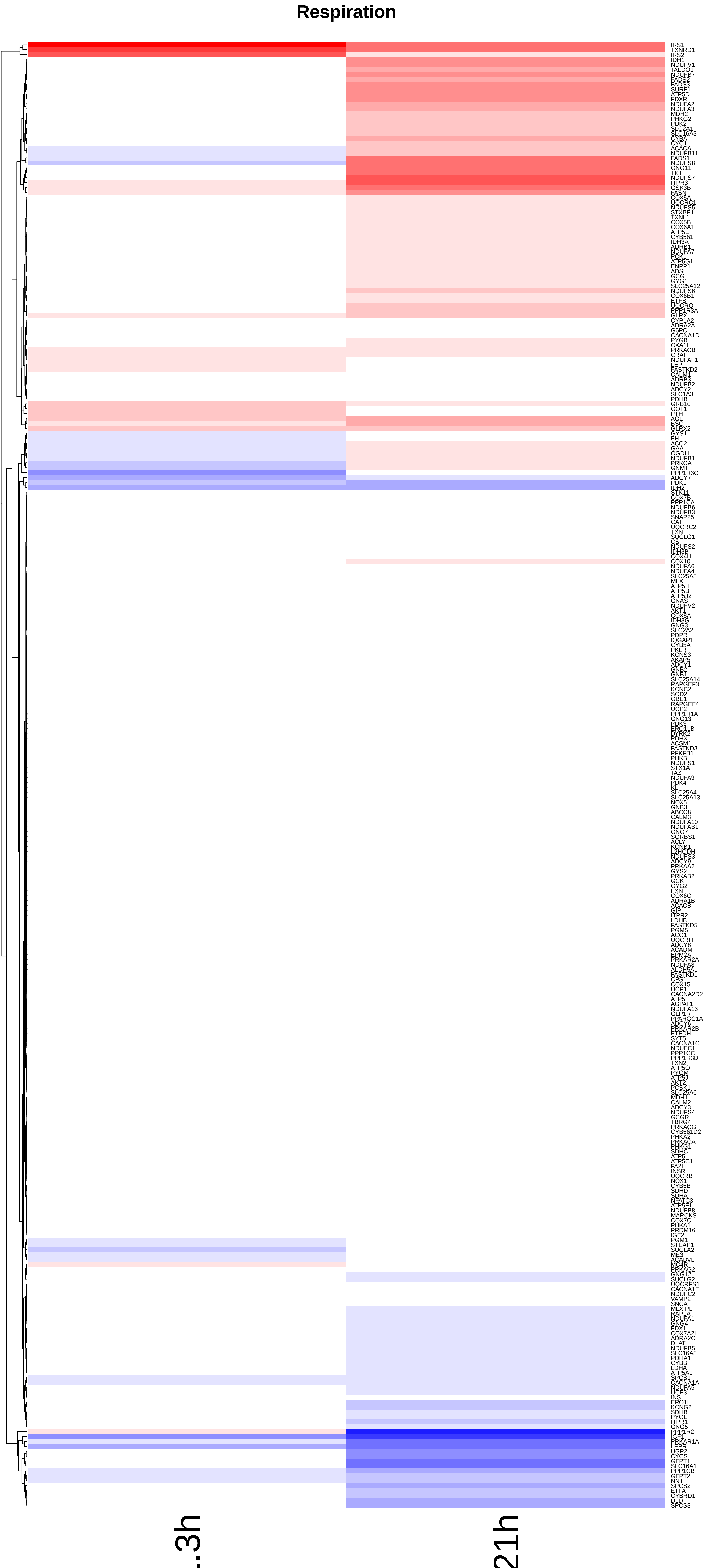

Respiration
..3h
21h
IRS1
TXNRD1
IRS2
IDH1
NDUFV1
TALDO1
NDUFB7
FADS2
FADS3
SURF1
ATP5D
FDXR
NDUFA2
NDUFA3
MDH2
PHKG2
PDK2
SLC2A1
SLC16A3
CYBA
CYC1
ACACA
NDUFB11
FADS1
NDUFS8
GNG11
TKT
NDUFS7
ITPR3
GSK3B
FASN
COX5A
UQCRC1
NDUFS5
STXBP1
TXNL1
COX5B
COX6A1
ATP5E
CYB561
IDH3A
ADRB1
NDUFA7
PCK1
ATP5G1
ENPP1
ADSL
GCG
GYG1
SLC25A12
NDUFS6
COX6B1
ETFB
UQCRQ
PPP1R3A
GLRX
CYP1A2
ADRA2A
G6PC
CACNA1D
PYGB
OXA1L
PRKACB
CRAT
NDUFAF1
LEP
FASTKD2
CALM1
ADRB3
NDUFB2
ADCY2
SLC1A3
PDHB
GRB10
GOT1
PTH
AGL
BSG
GLRX2
GYS1
FH
ACO2
GAA
OGDH
NDUFB1
PRKCA
GNMT
PPP1R3C
ADCY7
PDK1
IDH2
STK11
COX7B
PPP1CA
NDUFB6
NDUFB3
SNAP25
CAT
UQCRC2
TXN
SUCLG1
CS
NDUFS2
IDH3B
COX4I1
COX10
NDUFA6
NDUFA4
SLC25A5
MLX
ATP5H
ATP5B
ATP5J2
GNAS
NDUFV2
AKT1
COX8A
IDH3G
GNG3
SLC2A2
PDPR
IQGAP1
CYB5A
PKLR
KCNS3
AKAP5
ADCY1
GNB2
GNB1
SLC25A14
RAPGEF3
KCNC2
SOD2
GBE1
RAPGEF4
UCP2
PPP1R1A
GNG13
PDK3
ERO1LB
DYRK2
PDHX
ACSM1
FASTKD3
PFKFB1
PHKB
NDUFS1
STX1A
TAZ
NDUFA9
PDK4
KL
SLC25A4
SLC25A13
NOX5
GNB3
ABCC8
CALM3
NDUFA10
NDUFAB1
GNG7
SORBS1
ACLY
KCNB1
L2HGDH
NDUFS3
ADCY9
PRKAA2
GYS2
PRKAB2
GCK
GYG2
FXN
COX6C
ADRA1B
ACACB
GIP
ITPR2
LDHB
FASTKD5
PGM5
ACO1
UQCRH
ADCY8
ACADM
EPM2A
PRKAR2A
NDUFA8
ALDH5A1
FASTKD1
CPS1
COX15
UCP1
CACNA2D2
ATP5I
AGPAT1
NDUFA13
GLP1R
PPARGC1A
ADCY6
PRKAR2B
ETFDH
SYT5
CACNA1C
NDUFC1
PPP1CC
PPP1R3D
TXN2
ATP5O
PYGM
ATP5J
AKT2
PCSK1
SLC25A6
MDH1
CALM2
ADCY3
NDUFS4
GCGR
TBRG4
PRKACG
CYB561D2
PHKA2
PRKACA
PHKG1
SDHC
ATP5L
ATP5C1
FA2H
INSR
UQCRB
NOX1
CYB5B
SDHD
SDHA
NFATC3
ATP5F1
NDUFB8
MARCKS
COX7C
PHKA1
PRDM16
IGF2
PGM1
STEAP1
SUCLA2
ME3
ACADVL
MC4R
PRKAG2
GNG12
SUCLG2
UQCRFS1
CACNA1E
NDUFC2
VAMP2
SNCA
MLXIPL
RAP1A
NDUFA1
GNG4
FDX1
COX7A2L
ADRA2C
DLAT
NDUFB5
SLC16A8
PDHA1
CYBB
LDHA
ATP5A1
SPCS1
CACNA1A
NDUFA5
UCP3
INS
ERO1L
KCNG2
SDHB
PYGL
ITPR1
GNG5
PPP1R2
IGF1
PRKAR1A
LEPR
UGP2
CYCS
GFPT1
SLC16A1
PPP1CB
GFPT2
NNT
SPCS2
ETFA
CYBRD1
DLD
SPCS3

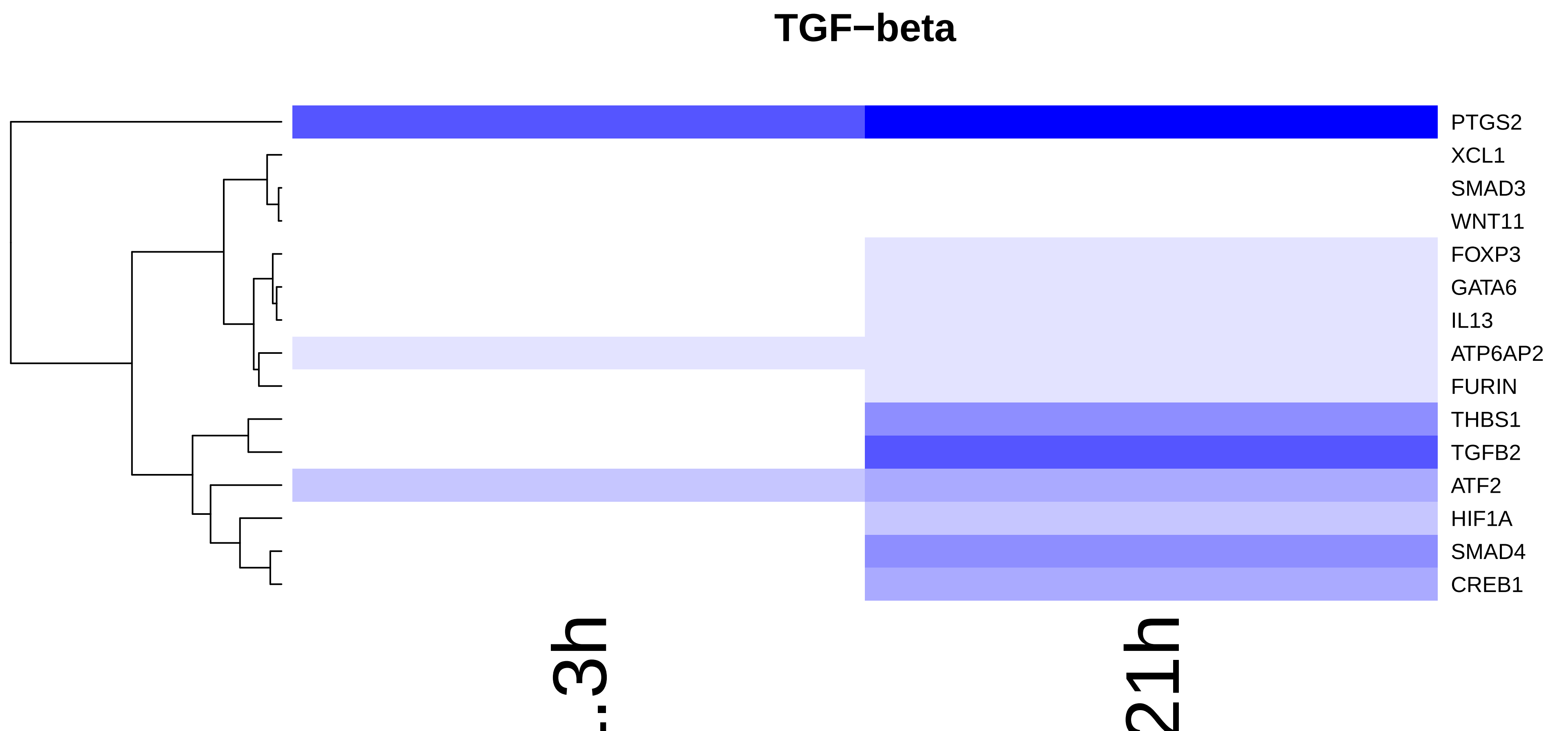

TGF−beta
PTGS2
XCL1
SMAD3
WNT11
FOXP3
GATA6
IL13
ATP6AP2
FURIN
THBS1
TGFB2
ATF2
HIF1A
SMAD4
CREB1

Translation

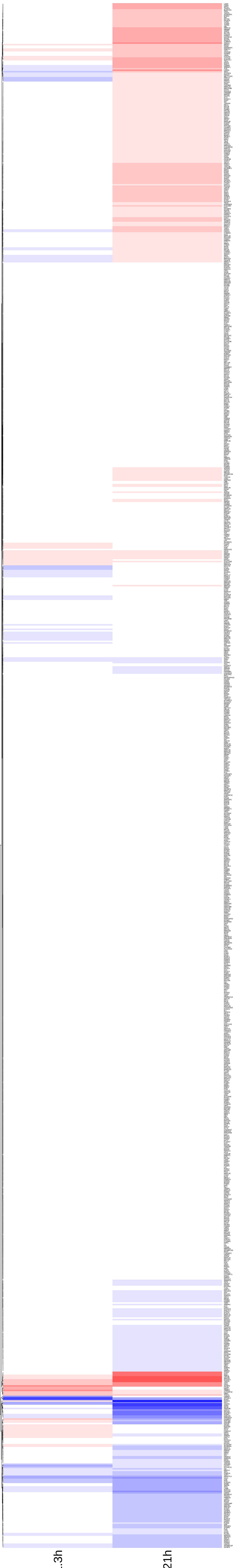

Supplement: Additional file 2 — Figure S1. Heat maps of SMC differential gene expression patterns, induced by treatment with moxLDL at 3h and 21h. Partial information from these heat maps was used to draw Figures 4, 6, 7 and 8. [file 1471-2261-13-4-S2.pdf]
